# Systemic neuroimmune responses in people with non-specific neck pain and cervical radiculopathy, and associations with clinical, psychological, and lifestyle factors

**DOI:** 10.3389/fnmol.2022.1003821

**Published:** 2022-10-13

**Authors:** Ivo J. Lutke Schipholt, Gwendolyne G. M. Scholten-Peeters, Meghan A. Koop, Petra Bonnet, Hetty J. Bontkes, Michel W. Coppieters

**Affiliations:** ^1^Department of Human Movement Sciences, Faculty of Behavioural and Movement Sciences, Amsterdam Movement Sciences, Vrije Universiteit Amsterdam, Amsterdam, Netherlands; ^2^Laboratory Medical Immunology, Department of Clinical Chemistry, Amsterdam University Medical Center, Amsterdam, Netherlands; ^3^Department of Rehabilitation Medicine, Amsterdam University Medical Centre, Amsterdam, Netherlands; ^4^Menzies Health Institute Queensland, Griffith University, Brisbane, QLD, Australia

**Keywords:** mononeuropathy, immunology, neuroscience, musculoskeletal health, spine, disc herniation, neck pain

## Abstract

Neuroimmune responses remain understudied in people with neck pain. This study aimed to (1) compare a broad range of systemic neuroimmune responses in people with non-specific neck pain (*N* = 112), cervical radiculopathy (*N* = 25), and healthy participants (*N* = 23); and (2) explore their associations with clinical, psychological and lifestyle factors. Quantification of systemic neuroimmune responses involved *ex vivo* serum and *in vitro* evoked-release levels of inflammatory markers, and characterization of white blood cell phenotypes. Inflammatory indices were calculated to obtain a measure of total immune status and were considered the main outcomes. Differences between groups were tested using analyses of covariance (ANCOVA) and multivariable regression models. Compared to healthy participants, the *ex vivo* pro-inflammatory index was increased in people with non-specific neck pain (β = 0.70, *p* = 0.004) and people with cervical radiculopathy (β = 0.64, *p* = 0.04). There was no difference between non-specific neck pain and cervical radiculopathy (β = 0.23, *p* = 0.36). Compared to non-specific neck pain, people with cervical radiculopathy showed lower numbers of monocytes (β = −59, *p* = 0.01). There were no differences between groups following *in vitro* whole blood stimulation (*p* ≥ 0.23) or other differences in the number and phenotype of white blood cells (*p* ≥ 0.07). The elevated *ex vivo* neuroimmune responses in people with non-specific neck pain and radiculopathy support the contention that these conditions encompass inflammatory components that can be measured systemically. There were multiple significant associations with clinical, psychological and lifestyle factors, such as pain intensity (β = 0.25) and anxiety (β = 0.23) in non-specific neck pain, visceral adipose tissue (β = 0.43) and magnification (β = 0.59) in cervical radiculopathy, and smoking (β = 0.59) and visceral adipose tissue (β = 0.52) in healthy participants. These associations were modified by sex, indicating different neuroimmune associations for females and males.

## Introduction

Non-specific neck pain and cervical radiculopathy are common and complex conditions with often a poor prognosis (Hush et al., 2011; Sleijser-Koehorst et al., 2018). A thorough understanding of the pathophysiology is currently lacking, but may involve a complex interplay between neuroimmune responses and clinical, psychological, and lifestyle factors (Chapman et al., 2008). A better understanding of neuroimmune responses in neck pain may reveal unknown mechanisms of persistent pain and might contribute to personalized and more effective therapies (Lasselin et al., 2016; Chimenti et al., 2018).

In recent years, research on the possible role of neuroimmune responses in people with persistent pain has gained significant interest (Sterling et al., 2013; Ji et al., 2016; Hore and Denk, 2019; Farrell et al., 2020; Sandy-Hindmarch et al., 2022). There is overwhelming evidence from preclinical studies that neuroimmune responses are central to the initiation, progression and resolution of persistent pain (Basbaum et al., 2009; Grace et al., 2021; Kavelaars and Heijnen, 2021; Parisien et al., 2022). Local immune activation can be found in people with neck pain, such as muscle and facet joint inflammation in traumatic neck pain, and within the intervertebral disc and nerve roots in people with a cervical radiculopathy (Abbed and Coumans, 2007; Kokubo et al., 2008; Aarnio et al., 2022). Besides local inflammation, an increasing body of evidence suggests the presence of enhanced circulating cytokines and enhanced evoked-release of inflammatory markers from circulating immune cells in persistent pain (Barbe and Barr, 2006; Teodorczyk-Injeyan et al., 2011; Sterling et al., 2013; Ethemoǧlu and Erkoç, 2020; Farrell et al., 2020; Zhou et al., 2021). A meta-analysis showed raised systemic *ex vivo* levels of inflammatory markers, including interleukin (IL)-1β, tumor necrosis factor (TNF)-α, and c-reactive protein (CRP) in non-specific neck pain (Farrell et al., 2020). The production of nociceptive chemokines c-c motif ligand 2 (CCL2) and CCL3, as well as the inflammatory markers TNF-α, IL-1β, and IL-6 were significantly increased after *in vitro* whole blood stimulation (Teodorczyk-Injeyan et al., 2011, 2015). Likewise, in cervical radiculopathy, a change in lymphocyte subsets and increased systemic CRP was found (Ethemoǧlu and Erkoç, 2020).

A key mediator in the initiation of systemic neuroimmune responses are Toll-like receptors (TLRs) which are capable of recognizing endogenous and exogenous danger (Lacagnina et al., 2018). Activation of TLRs may result in the production of inflammatory markers and the generation of hyperexcitable sensory neurons and thereby participate in neural signaling associated with pain states (Austin and Fiore, 2019; Liu et al., 2021). It is speculated that in people with persistent pain, immunocompetent cells can be sensitized and may produce an exaggerated response following subsequent exposure to a danger stimulus (Kwok et al., 2013). A long list of potential endogenous and exogenous factors contributing to enhanced systemic neuroimmune responses has been identified, such as clinical (e.g., disability), lifestyle [e.g., body mass index (BMI)], and psychological (e.g., rumination) factors (Lutke Schipholt et al., 2018; Rogero and Calder, 2018; Furman et al., 2019; Wang et al., 2020). These factors may produce danger signals [e.g., associated molecular patterns (Moseley and Butler, 2017)] and subsequently the activation of TLR pathways (Bruno et al., 2018; Nie et al., 2018).

Despite the increased interest in neuroimmune responses in neck pain, previous studies only evaluated a limited subset of inflammatory markers (Teodorczyk-Injeyan et al., 2011, 2019; Kwok et al., 2012, 2013), did not investigate associations between neuroimmune responses and psychological or lifestyle factors (Teodorczyk-Injeyan et al., 2011, 2019; Kwok et al., 2012, 2013; Farrell et al., 2020), and used diverse preanalytical and analytical methods [e.g., either whole-blood cultures or peripheral blood mononuclear cell (PBMC) stimulation] (Teodorczyk-Injeyan et al., 2011, 2019; Kwok et al., 2012, 2013; Farrell et al., 2020). These studies provide an incomplete picture of the neuroimmune responses in people with neck pain. Therefore, we conducted a cross-sectional study which evaluated a broad range of neuroimmune responses in people with and without neck pain. This study aimed to (1) compare systemic neuroimmune responses between people with non-specific neck pain, cervical radiculopathy and healthy participants, and (2) to study the associations between neuroimmune responses and clinical, psychological and lifestyle factors.

## Materials and methods

This manuscript followed the STROBE guidelines for cross-sectional studies (von Elm et al., 2007). Ethical approval was obtained by the Medical Ethics Committee of Amsterdam University Medical Centre, location VUmc (approval number: 2018.181; approval date: 27-12-2018) and was registered at trialregister.nl (study ID: NL6575). All participants signed a written informed consent prior to participating.

### Participants

People with non-specific neck pain (Haldeman et al., 2008) and people with cervical radiculopathy were recruited from general medical practices, primary care physical therapy clinics and outpatient neurology and orthopedic departments of secondary care hospitals, during their initial consultation (i.e., before treatment had commenced). The diagnosis cervical radiculopathy was based on clinical signs and symptoms [including radicular pain (Finnerup et al., 2016)] that concurred with nerve root compression identified *via* MRI. The clinical diagnosis was made by a medical specialist and confirmed with MRI which had to show nerve compression at a relevant level. The symptom duration of non-specific neck pain and cervical radiculopathy had to be at least 6 weeks. Healthy participants, who had to be free of musculoskeletal pain for a minimum of 3 months, were recruited from the general population. All participants had to be aged between 18 and 65 years and needed to have a sufficient speaking and reading level of the Dutch language to participate in the trial. Exclusion criteria for all groups were: pregnancy or less than 9 months postpartum, contraindications for venipuncture (e.g., phlebitis), use of corticosteroids or cytokine modulatory medication (e.g., infliximab) in the preceding 6 weeks, use of botulinum toxin (Botox) injection during the preceding 3 months, non-steroid anti-inflammatory medication use within the past week, long-distance flight within the past week, known comorbidities with immune/endocrine involvement (e.g., ankylosing spondylitis and rheumatoid arthritis), medical red flags suggestive of serious pathology (Bier et al., 2018; Finucane et al., 2020) or a diagnosed psychological or psychiatric condition (e.g., clinical depression).

### Preparation of blood samples

Following the initial screening, heparinized and clot activated samples of peripheral blood (7 ml each) were obtained by venipuncture from the antecubital fossa. Fasting blood samples were obtained between 08:00 and 09:00 a.m. and were processed within 4-h.

Heparinized samples of peripheral blood were used for whole blood culture. To induce the production of inflammatory mediators, whole blood cultures were cultivated for 24 h at 37°C in a humified 5% CO_2_ incubator with lipopolysaccharide (TLR4 stimulation) from *Escherichia coli* serotype 055:B5 (LPS; Sigma) at concentrations of 10 μg/ml [high dose (HD-LPS)] and 1 ng/ml [low dose (LD-LPS)]. Following the incubation period, supernatants were centrifuged, aliquoted, and frozen at −80°C until analyzed. Aliquots of blood samples to determine serum *ex vivo* levels of inflammatory markers were stored at −80°C after centrifugation for 10 min at 1,530*g*.

### Measurement of neuroimmune responses

A broad range of neuroimmune responses was assessed: (a) serum *ex vivo* levels of inflammatory markers, (b) levels of inflammatory markers following *in vitro* stimulation of whole blood, and (c) phenotypic analysis of white blood cells (Table 1).

**TABLE 1:** Included neuroimmune markers.

Domain	Neuroimmune parameters
Systemic inflammatory marker directly from blood samples *(ex vivo*)^a^	TNF-α (0.30–1,160), sTNF-R2 (1.29–2,150), IL-1β (0.16–1,530), IL-1RA (7.37–4,500), hsCRP^b^
Inflammatory marker concentration after *in vitro* stimulation of whole blood cells^c^	TNF-α (0.04–248), IL-1β (0.04–375), IL-1RA (1.12–650), IL-4 (0.02–158), IL-10 (0.14–3,700), CCL2 (0.09–375), CCL3 (3.02–743), CCL4 (0.37–750)
Phenotypic analysis of white blood cells cells^d^	CD45^+^, CD3^+^, CD4^+^, CD25^hi^, CD8^+^, CD56^+^, CD19^+^, CD14^+^, HLA-DR, TLR4

The values between brackets indicate the lower limit of quantification and upper limit of quantification (LLOQ-ULOQ). ^a^Measured using multianalyte assay Ella (R&D systems, Minneapolis, MN, USA). Inter-assay coefficient of variation: TNF-α 4.27%, sTNF-R2 5.78%, IL-1β 4.97, IL-1RA 7.20%. ^b^Cardiac C-reactive protein (Latex) high sensitive using Roche/Hitachi cobas c systems. ^c^Stimulated for 24 h at 37°C, in a humidified 5% CO_2_ incubator, with lipopolysaccharide (LPS) from Escherichia coli O55:B5 at a concentration of 1 ng/ml (LD-LPS) and 10 μg/ml (HD-LPS). Determined using a custom-made U-plex (MSD, Maryland, MD, USA). Supernatant were diluted 100-fold a prior testing for the inflammatory marker of interest. Inter-assay coefficient of variation: TNF-α 7%, IL-1β 12.7%, IL-1RA 10.6%, IL-10 22%, CCL2, 8.4%, CCL3 12.7%, CCL4 12.9%. ^d^Determined by 10-color flow cytometry (FCM): CD45^+^, General Leukocyte marker; CD3^+^, T-cell marker; CD3^+^ CD4^+^, CD4^+^ T-helper marker; CD3^+^ CD4^+^ CD25hi, T-regulator cell marker; CD3^+^ CD8^+^, Cytotoxic T-cell marker; CD3-CD56^+^, Natural Killer cell marker; CD19^+^, B-cell marker; CD14^+^, monocyte marker; HLA-DR, activation marker for T-cells and monocytes; CD25^+^, activation marker for T-cells; TLR4, Toll-like receptor 4 marker. TNF-α: tumor necrosis factor-α; sTNF-R2, tumor necrosis factor receptor antagonist 2; IL-1β, interleukin-1β; IL-1RA, interleukin-1 receptor antagonist; hsCRP, high sensitive C-reactive protein; IL-4, interleukin-4; IL-10, interleukin-10; CCL2, c-c-motif chemokine ligand 2; CCL3, c-c-motif chemokine ligand 3; CCL4, c-c-motif chemokine ligand 4; CD, cluster of differentiation.

#### *Ex vivo* serum and *in vitro* whole blood evoked-release levels of inflammatory markers

The *ex vivo* levels of TNF-α, IL-1β, sTNFR-R2, and IL-1RA were measured using multianalyte assay ELLA (R&D systems, Minneapolis, MN, USA) and hsCRP using (Roche/Hitachi cobas c systems). The *in vitro* evoked-release of TNF-α, IL-1β, IL-10, IL-4, IL-1RA, CCL2, CCL3, and CCL4 were determined using a custom-made U-plex (MSD, Maryland, MD, USA). Supernatants were diluted 100-fold. In case the inflammatory marker level was below the lower limit of quantification (LLOQ) the value was substituted with half the LLOQ value (Croghan and Egeghy, 2003). These inflammatory markers were selected as these have been shown to be related to musculoskeletal pain (Barbe and Barr, 2006; Teodorczyk-Injeyan et al., 2011; Sterling et al., 2013; Ethemoǧlu and Erkoç, 2020; Farrell et al., 2020; Zhou et al., 2021).

#### Characterization of white blood cell phenotypes

Fluorescence-activated cell sorting staining was used for cell surface staining of mononuclear cells using a whole blood staining protocol and red blood cell lysis using optilyse B, conform manufacturer recommendations (Beckman Coulter, Brea, CA, USA). In short, 50 μL of Optilyse B Lysing Solution was added per tube containing 50 μl of whole blood, incubated at room temperature for 10 min. Thereafter, 500 μL of deionized water was added followed by brief vortex mixing and followed by flow cytometry preparations after 10 min. To quantify lymphocyte subsets, Trucount tubes were used (BD Biosciences, Franklin Lakes, NJ, USA). The following monoclonal antibodies were used: CD8-APC-AF700 (B9.11), CD19-ECD (J3-119), CD56-PC7 (N901; all from Beckman Coulter, Brea, CA, USA); HLA-DR-FITC (G46-6), CD14-APC (M5E2), TLR4-PE (TF901; all from BD Pharmingen, San Diego, CA, USA) and CD3-APC (SK7), CD4-APC-H7 (SK3), CD25-PE (2A3), CD45-PerCP (2D1; all from BD Biosciences, Franklin Lakes, NJ, USA) (see Table 1). HLA-DR was used as activation marker for T-cells and monocytes, CD25 was used as activation marker for T-cells; TLR-4 expression was assessed on monocytes. Isotypes were used as control for these activation markers. Samples were run on fluorescence-activated cell sorting Gallios (Beckman Coulter, Brea, CA, USA) and analyzed using Kaluza (Beckman Coulter, Brea, CA, USA). The total number of leucocytes was determined using Z2 analyzer (Beckman Coulter, Brea, CA, USA). In Supplementary material A provides more information about the gating strategy for the flow cytometry analysis.

### Questionnaires and measurements

General health and demographic data, including age, smoking history, co-morbidities, and medication use at the time of the study were collected. Additionally, several clinical, psychological, and lifestyle questionnaires were used to evaluate sleep quality, physical activity, pain catastrophizing, central sensitization, disability, general mental health, and neuropathic pain. Table 2 provides an overview of all questionnaires. As psychological discomfort without the presence of an actual clinical psychological condition is often present in people with persistent pain, we also evaluated depressive, anxiety, and distress symptoms (Linton and Shaw, 2011). Visceral adipose tissue was assessed as the linear distance between abdominal peritoneum and the ventral aspect of vertebrae T12 using ultrasound imaging (Philips ClearVue 550; C5-2, range 5–2 MHz Convex, Eindhoven, Netherlands). These factors were selected as they are all risk factors and/or associated with inflammatory markers (Supplementary material B; Abramson and Vaccarino, 2002; Generaal et al., 2014; Matute Wilander et al., 2014; Shiels et al., 2014; Schlecht et al., 2016; Klyne et al., 2017, 2021; Fitzcharles et al., 2021; Gregus et al., 2021; Koop et al., 2021).

**TABLE 2:** Questionnaires and measures.

Domain	Description and scoring
Co-morbidities	Number of co-morbidities (e.g., high blood pressure)
Alcohol use	Non-drinker/drinker
Smoking	Never smoked/former smoker/current smoker
Body mass index (BMI)	BMI self-reported body weight (kg) divided by height (m^2^)
Medication use	Number of medications used
Age	Age in years
Psychological status (MHI-5)	The MHI-5 consist of five statements regarding psychological wellbeing. The final MHI-5 score is calculated by summing up the item scores and transforming this score to a scale varying from 0 to 100, with lower scores indicating more severe depressive symptoms (Cuijpers et al., 2009).
Physical activity (IPAQ)	International Physical Activity Questionnaire, expressed in 1,000 metabolic equivalent minutes per week (Dutch version) (Hallal and Victora, 2004).
Disability (NDI)	The Dutch version of the NDI is a valid and responsive measure of disability. Each section is scored on a 0 to 5 rating scale, in which zero means “No pain” and 5 means “Worst imaginable pain.” 0% means: no activity limitations, 100% means complete activity limitation (Jorritsma et al., 2012).
Fear of movement (TAMPA-24)	Preferred self-administrated questionnaire to asses fear of movement in musculoskeletal pain. A total score of >37 is considered as kinesiophobia (Sleijser-Koehorst et al., 2019).
Type of pain (PD-Q)	Persistent pain will be categorized in two-mechanism based groups: nociceptive and neuropathic pain using the PDQ. The PD-Q is a reliable screening tool with high specificity (Freynhagen et al., 2006).
Type of pain (CSI)	The Dutch Central Sensitization Inventory (CSI) has good internal consistency, good discriminative power and excellent test-retest reliability. A cut-off score of 40/100 provides a sensitivity of 81% and specificity of 75% (Neblett et al., 2013).
Depression, anxiety, stress (DASS-21)	Preferred self-administrated questionnaire to assess depression, anxiety, and stress in musculoskeletal pain (Bijker et al., 2019; Sleijser-Koehorst et al., 2019).
Rumination, magnification, helplessness (PCS)	Preferred self-administrated questionnaire to assess pain catastrophizing in musculoskeletal pain (Sleijser-Koehorst et al., 2019).
Sleep quality, sleep duration (PSQI)	Total score above 5 yield a sensitivity of 89.6% and specificity of 86.5% in distinguishing good and poor sleepers (Buysse et al., 1989).
Pain intensity (VAS)	The visual analog scale (VAS) is a validated, subjective measure for acute and chronic pain. Scores are recorded by making a mark on a 10-cm line that represents a continuum between “no pain” and “worst pain.”
Visceral adipose tissue (VAT)	Linear distance between abdominal peritoneum and ventral aspect of vertebrae will be assessed using ultrasonography (Park et al., 2005; Schlecht et al., 2014).

BMI, body mass index; MHI-5, mental health inventory-5; IPAQ, international physical activity questionnaire; NDI, neck disability index; TAMPA, Tampa scale of kinesiophobia; PD-Q, pain detect questionnaire; CSI, central sensitization questionnaire; DASS21, depression, anxiety, stress questionnaire; PCS, pain catastrophizing questionnaire; PSQI, Pittsburgh sleep quality index; VAS, visual analog scale.

### Inflammatory indices

As summary measures, we calculated an inflammatory, pro-inflammatory, and anti-inflammatory index, for both *in vitro* and *ex vivo* levels, and the ratios between pro- and anti-inflammatory indices (Lutke Schipholt et al., 2022). This reduced the number of main analyses substantially. The inflammatory index was used as a summary measure which contains all inflammatory markers. The pro-inflammatory index was calculated based on the pro-inflammatory markers and the anti-inflammatory index was based on the anti-inflammatory markers. For exploration, individual inflammation markers were analyzed as secondary outcomes (Generaal et al., 2014). Inflammatory indices were calculated as the sum of all Ln-transformed (to create normal distributed data) *z*-standardized markers divided by the total number of markers used (Lutke Schipholt et al., 2022). *Z*-transformation of each marker was based on the healthy participant data. Consequently, positive *z*-scores indicate an increase in neuroimmune response, and negative *z*-scores indicate a reduced neuroimmune response. The individual *z*-score was calculated as: $\frac{\text{value}\,\mathrm{individual}\,\mathrm{patient}-\;\mathrm{mean}\;\left(\mathrm{healthy}\;\mathrm{group}\right)}{\text{SD}\;\left(\mathrm{healthy}\;\mathrm{group}\right)}$. To determine the association between the inflammatory indices and the clinical, psychological and lifestyle factors, the following *z*-score calculations were used: $\frac{\mathrm{value}\;\mathrm{individual}-\mathrm{mean}\,\left(\mathrm{group}\right)}{\text{SD}\,\left(\mathrm{group}\right)}$. The following calculations were used to determine the separate indices *ex vivo*: inflammatory index $=\frac{\text{zTNF}\alpha +\text{zTNFR}2+\text{zIL}\,1\,\beta +\text{zIL}\,1\,\text{RA}+\text{zhsCRP}}{5}$; pro inflammatory index $=\frac{\text{zTNF}\,\alpha +\;\mathrm{zIL1}\,\beta +\;\mathrm{zhsCRP}}{3}$; $\mathrm{anti}\,\mathrm{inflammatory}\,\mathrm{index}=\frac{\text{zTNFR}\,2+\text{zIL}\,1\,\text{RA}}{2}$ and *in vitro:* $\mathrm{inflammatory}\,\mathrm{index}=\frac{\text{TNF}\,\alpha +\text{zIL}\,1\,\beta +\text{zIL}\,1\,\text{RA}+\text{zIL}\,4+\text{zIL}\,10+\text{zCCL}\,2+\text{zCCL}\,3+\text{zCCL}\,4+\text{zhsCRP}}{9}$; pro inflammatory index $=\frac{\text{zTNF}\,\alpha +\text{zIL}\,1\,\beta +\text{zCCL}\,2+\text{zCCL}\,3+\text{zCCL}\,4+\text{zhsCRP}}{6}$; $\mathrm{anti}\,\mathrm{inflammatory}\,\mathrm{index}=\frac{\text{zIL}\,1\,\text{RA}+\text{zIL}\,4+\text{zIL}\,10}{3}$. The ratio inflammatory index was calculated as ratio pro anti inflammatory index $=\frac{\mathrm{pro}\;\mathrm{inflammatory}\;\mathrm{index}}{\mathrm{anti}\;\mathrm{inflammatory}\;\mathrm{index}}$.

### Sample size

Based on analyses of covariance (ANCOVA), three groups, an α of 0.05, β of 0.8, a sample size of 25 in each group had 80% power to detect a difference in means assuming a large effect size (*d* = 0.54) with sample failure of 15% for *in vitro* levels of TNF-α, and plate number and stimulation time as covariate.

### Statistical analyses

Descriptive characteristics are reported as means, median or percentages for the three groups of participants. For the examination of differences between the groups, analysis of variance (ANOVA) was conducted to determine a statistically significant difference on general health, demographic data, questionnaires, and physical tests. If data were not-normally distributed or were categorical, the Pearson Chi-square or independent sample Kruskall–Wallis was used. ANCOVA were used to test for differences in *ex vivo* and evoked-release *in vitro* levels of inflammatory markers, and characterization of white blood cell phenotypes. Data that were not normally distributed as indicated by the Kolmogorov–Smirnov test (*p* < 0.05) and visual inspection of the histogram, were Ln-transformed. The systemic markers directly from blood samples were adjusted for plate number. For the *in vitro* stimulation of whole blood cells two models were tested: (1) adjusted for plate number, LPS lot number and time between blood withdrawal and stimulation, (2) normalized (/1,000 monocytes) and adjusted for plate number, LPS lot number and time between blood withdrawal and stimulation.

Multivariable linear regression and linear regression were used to express the differences between groups in unstandardized beta-coefficient (B). The associations between neuroimmune responses and clinical, psychological and lifestyle factors were expressed as the standardized coefficient-β and standard error (SE). For all statistical tests, a *p*-value of less than 0.05 was regarded as significant, and interaction was examined for sex. All statistical analyses were performed using SPSS, version 28 (IBM, Armonk, NY, USA).

## Results

### Demographic and clinical variables

People with non-specific neck pain (*n* = 134), cervical radiculopathy (*n* = 36), and healthy participants (*n* = 26) were assessed for eligibility criteria, of whom 112 people with non-specific neck pain, 25 with cervical radiculopathy and 23 healthy participants were included in the study. The flow diagram of the study is presented in Supplementary material C. The people with non-specific neck pain in the present study were also part of another study with identical selection criteria to investigate the effects of joint mobilization and manipulation on neuroimmune responses in non-specific neck pain (Lutke Schipholt et al., 2022). For that study, 112 people with non-specific neck pain participated. Although only 25 participants were required, we decided to include all 112 people in the current study. Both patient groups demonstrated moderate pain, disability, sleep disturbances, and modest psychological distress scores compared to the healthy participants (Table 3). Most people with cervical radiculopathy had C6 (*n* = 11) or C7 (*n* = 10) nerve root involvement; a few had C5 (*n* = 1), C5 and C6 (*n* = 1), or C6 and C7 (*n* = 2) involvement.

**TABLE 3:** Demographic and clinical characteristics.

	Healthy participants Group A	Non-specific neck pain Group B	Cervical radiculopathy Group C	*P*-value Group A vs. Group B	*P*-value Group A vs. Group C	*P*-value Group B vs. Group C	*P*-value overall
**Characteristic**							
Age	49 (25)	49 (23)	46 (19)	0.93^†^	0.93^†^	0.93^†^	0.93^†^
Sex (male), %	39	33	36	0.84^‡^	0.84^‡^	0.81^‡^	
BMI, kg/m^2^	24 (4.2)	26 (4.4)	26 (4.7)	0.11^†^	0.11^†^	0.11^†^	0.11^†^
Pain intensity (VAS)	0 (0)	51 (19)	57 (13)	<0.001^†^	<0.001^†^	0.89^†^	<0.001^†^
Duration symptoms in weeks*	0 (0–0)	36 (10–120)	36 (13–260)	<0.001^§^	<0.001^§^	0.38^§^	<0.001^§^
Current smoker, %	9	29	44	0.11^‡^	0.11^‡^	0.29^‡^	
Former smoker, %	35	26	24	0.11^‡^	0.11^‡^	0.29^‡^	
Alcohol use, %	82	40	64	0.008^‡^	0.008^‡^	0.89^‡^	
Disability (NDI)*	2 (0–18)	30 (13–50)	32 (22–59)	0.001^§^	0.001^§^	0.64^§^	<0.001^§^
Neuropathic pain, %	0	12	52	<0.001^‡^	<0.001^‡^	<0.001^‡^	
Depressive symptoms (DASS21)*	0 (0–4)	4 (0–22)	4 (0–25)	<0.001^§^	0.002^§^	0.73^§^	<0.001^§^
Anxiety symptoms (DASS21)*	0 (0–5)	4 (1–18)	4 (0–22)	0.01^§^	<0.001^§^	0.70^§^	<0.001^§^
Stress symptoms (DASS21)*	0 (0–15)	8 (3–22)	6 (1–28)	0.01^§^	0.19^§^	0.57^§^	0.01^§^
Pain catastrophizing (total score)*	5 (0–26)	15 (9–23)	17 (10–30)	<0.001^§^	0.002^§^	0.87^§^	<0.001^§^
Pain rumination*	1 (0–7)	6 (4–10)	7 (3–12)	0.002^§^	0.01^§^	0.87^§^	0.007^§^
Pain magnification*	1 (0–2)	2 (1–4)	3 (0–5)	0.07^§^	0.07^§^	0.07^§^	0.07^§^
Pain helplessness*	1 (0–8)	8 (4–16)	8 (3.5–12)	<0.001^§^	0.001^§^	0.87^§^	<0.001^§^
Central sensitization (CSI > 40), %	8	40	40	0.06^‡^	0.06^‡^	0.89^‡^	
Mental health (MHI-5)*	80 (76–92)	73 (64–92)	76 (68–92)	0.004^§^	0.27^§^	0.09^§^	0.005^§^
Sleep quality (PSQI > 5), %	13	33	28	0.001^‡^	<0.001^‡^	0.63^‡^	
Kinesiophobia (Tampa > 37), %	4.5	28	28	0.05^‡^	0.05^‡^	0.97^‡^	
Physical activity (IPAQ)*	11.2 (6–15)	10 (7–16.7)	8.4 (8–17)	0.37^§^	0.37^§^	0.37^§^	0.37^§^
Visceral adipose tissue (mm)*	44 (30–69)	62 (45–79)	61 (38–79)	0.03^§^	0.25^§^	0.72^§^	0.04^§^
No comorbidity, %	68	36	40	0.01^‡^	0.08^‡^	0.33^‡^	
High blood pressure, %	21	13	8	0.11^‡^	0.15^‡^	0.68^‡^	
Other MSK pain, %	0	50	20	<0.001^‡^	<0.001^‡^	0.04^‡^	
Acetaminophen use, %	0	5	12	0.31^‡^	0.31^‡^	0.31^‡^	
Diuretics use, %	8	7	8	0.95^‡^	0.95^‡^	0.95^‡^	
Anticonception, %	13	11	12	0.97^‡^	0.97^‡^	0.97^‡^	
Proton pump inhibiter, %	4	8	8	0.53^‡^	0.53^‡^	0.53^‡^	
Anticoagulantia, %	8	7	0	0.43^‡^	0.43^‡^	0.43^‡^	

Values are presented as mean (SD) for continuous data and as percentages for categorical data unless stated otherwise. *Data represented as median and interquartile range (25th–75th percentiles). ^†^Analysis of variance (ANOVA). ^‡^Pearson Chi-square test. ^§^Independent sample Kruskall–Wallis. BMI, body mass index; VAS, visual analog scale (0–100); NDI, neck disability index (0–100); DASS21, depression, anxiety, stress score; MHI-5, mental health inventory-5; IPAQ presented in 1,000 METs; mm, millimeter; MSK, musculoskeletal.

### Comparison of neuroimmune responses between groups

#### *Ex vivo* serum inflammatory marker concentrations

**FIGURE 1:**
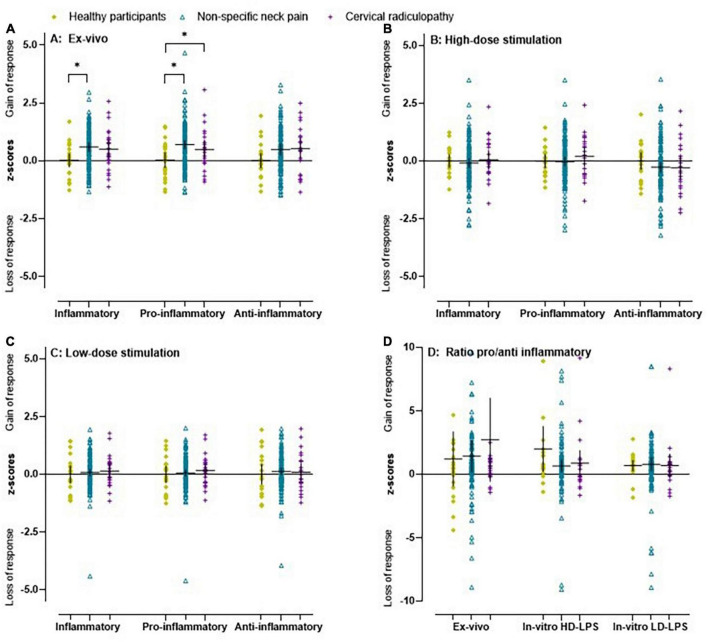
Scatter plot and mean (95% CI) of *z*-scores for inflammatory indices in people with non-specific neck pain, cervical radiculopathy and healthy participants. **(A)** *Ex vivo* inflammatory, pro-inflammatory, and anti-inflammatory index in healthy participants, people with non-specific neck pain and cervical radiculopathy. **(B)** The *in vitro* inflammatory, pro-inflammatory, and anti-inflammatory indices after whole blood stimulation with TLR4 agonist lipopolysaccharide at a concentration of 10 μg/ml (high dose LPS) in healthy participants, people with non-specific neck pain and cervical radiculopathy. The *in vitro* values were first/1,000 monocytes normalized, Ln-transformed, and finally *z*-score standardized. **(C)** Inflammatory indices at a concentration of 1 ng/ml (low dose LPS) in healthy participants, people with non-specific neck pain and cervical radiculopathy. **(D)** The ratio between the *in vitro* 10 μg/ml, *in vitro* 1 ng/ml, and *ex vivo* pro-inflammatory and anti-inflammatory index in healthy participants, people with non-specific neck pain and cervical radiculopathy. Lines represent mean (*horizontal line*), and 95% CI. The scatter plot was based on the individual points of 112 people with non-specific neck pain, 25 with cervical radiculopathy and 23 healthy participants. *Represents a significant difference between groups (*p* < 0.05).

The unadjusted and adjusted *ex vivo* inflammatory index (β = 0.54) and *ex vivo* pro-inflammatory index (β = 0.70) were significantly higher in the non-specific neck pain group compared to healthy participants (Figure 1A and Supplementary material D). For the cervical radiculopathy group, the unadjusted *ex vivo* pro-inflammatory index (β = 0.64) was significantly elevated compared to the healthy participants (Figure 1A and Supplementary material D). No differences were observed between people with non-specific neck pain and cervical radiculopathy. In the people with non-specific neck pain and cervical radiculopathy, we found effect modification based on sex (Supplementary material D).

**FIGURE 2:**
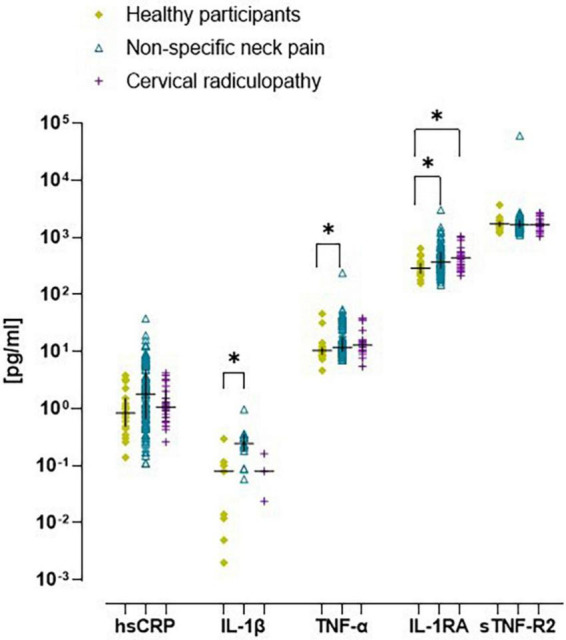
Scatter plot and median (inter quartile range) of *ex vivo* concentration of inflammatory markers in healthy participants, people with non-specific neck pain, and cervical radiculopathy. Raw *ex vivo* data were Ln-transformed before testing for significant differences between groups. Lines represent median (*horizontal line*), and 25th–75th percentiles. hsCRP, high sensitive – c reactive protein; TNF-α, tumor necrosis factor-α; IL-1β, interleukin-1β; IL-1RA, interleukin one-receptor antagonist; sTNF-R2, tumor necrosis factor – receptor two. *p < 0.05. The scatter plot was based on the individual points of 112 people with non-specific neck pain, 25 with cervical radiculopathy and 23 healthy participants.

All inflammatory markers were Ln-transformed to normalize the residual variances. There was a significant increase in prevalence of low-grade inflammation (hsCRP > 3 mg/L) in people with non-specific neck pain (32%; *p* = 0.04), but not with cervical radiculopathy (20%; *p* = 0.70) compared to healthy participants (13%). The *ex vivo* levels of IL-1β were generally below the limit of detection, but were measurable in approximately ∼40% of the cases. In the people with non-specific neck pain, the *ex vivo* concentrations of IL-1β, TNF-α, and IL-1RA were significantly elevated compared to healthy participants (Figure 2 and Supplementary material D). People with cervical radiculopathy showed significantly elevated *ex vivo* levels of IL-1RA, a trend for increased hsCRP (*p* = 0.06), and no significant difference in IL-1β, TNF-α, and sTNF-R2 compared to healthy participants (Figure 2 and Supplementary material D). There were no differences in inflammatory markers between people with non-specific neck pain and cervical radiculopathy.

#### Phenotypic analysis of white blood cells

The phenotypic analysis of white blood cells revealed lower levels of monocytes (β = −59, *p* = 0.01) in the cervical radiculopathy group compared to the non-specific neck pain group (Table 4). There were no other significant differences in activation markers of PBMCs between groups. There was no effect modification by sex.

**TABLE 4:** Phenotypic analysis of peripheral white blood cells in people with non-specific neck pain, people with a cervical radiculopathy and healthy participants.

PBMC phenotype	Healthy participants [mean (SD)]	People with non-specific neck pain [mean (SD)]	People with cervical radiculopathy [mean (SD)]	*P*-value
Leukocytes^1^	6.7 (1.7)	6.3 (1.8)	6.3 (1.5)	0.53^†^
PBMC^2^	2982 (722)	2749 (904)	2545 (646)	0.21^†^
Lymphocytes^2^	2537 (612)	2392 (855)	2210 (638)	0.36^†^
Monocytes^2^	286 (129)	273 (111)	**214 (71)**	0.03^†^ 0.07^‡^ 0.04^#^ 0.99^§^
B-cells^2^	283 (120)	305 (187)	238 (122)	0.21^†^
% B-cells/lymphocytes	10.8 (3.9)	12.5 (5.4)	10.6 (4.1)	0.12^†^
NK-cells^1^	214 (173)	221 (168)	262 (148)	0.49^†^
% NK-cells/lymphocytes	8.4 (6.1)	9.3 (4.8)	11.7 (5.7)	0.06^†^
T-cells^2^	1983 (565)	1811 (721)	1632 (499)	0.20^†^
% T-cells/lymphocytes	78.2 (8.9)	75.3 (7.6)	74.2 (7.2)	0.17^†^
CD4^+^ T-cells^2^	1283 (433)	1174 (505)	1012 (354)	0.14^†^
% CD4^+^ T-cells	65.5 (13.7)	65.4 (10.3)	62.4 (10.4)	0.46^†^
CD8^+^ T-cells^2^	592 (407)	545 (293)	513 (263)	0.68^†^
% CD8^+^ T-cells	29.1 (13.1)	29.6 (9.7)	31.2 (9.7)	0.75^†^
% DNT/T-cells	3.9 (2.5)	4.0 (2.8)	5.4 (3.4)	0.06^†^
% DPT/T-cells	1.4 (2.9)	1.6 (6.1)	0.83 (0.63)	0.81^†^
% CD56^+^ CD3^+^ T-cells	4.4 (5.6)	3.9 (4.2)	5.2 (10.8)	0.59^†^
% T-reg/CD4^+^ T-cells	7.2 (2.2)	7.1 (1.9)	7.2 (1.6)	0.95^†^
% CD25^+^ CD4^+^ T-cells	39.3 (11.9)	44.5 (15.6)	46.7 (13.5)	0.20^†^
% CD25^+^ CD8^+^ T-cells	10.6 (8.9)	13.0 (9.8)	14.1 (11.1)	0.44^†^
% HLA-DR^+^ T-cells	5.9 (6.1)	6.4 (5.0)	6.1 (4.7)	0.95^†^
% HLA-DR^+^ CD4^+^ T-cells/CD4^+^ T-cells	4.1 (3.6)	4.3 (2.9)	4.2 (2.7)	0.95^†^
% HLA-DR^+^ CD8^+^ T-cells/CD8^+^ T-cells	8.1 (8.8)	10.2 (9.2)	9.1 (7.9)	0.55^†^
% CD14^+^/PBMC	7.6 (3.4)	8.7 (3.7)	6.9 (3.9)	0.06^†^
TLR4^+^ monocytes/CD14^+^ ^1^	0.20 (0.06)	0.24 (0.09)	020 (0.05)	0.06^†^
HLA-DR^+^ monocytes/CD14^+ 1^	0.06 (0.03)	0.06 (0.03)	0.06 (0.02)	0.48^†^

^1^Absolute number: × 10^9^/L. ^2^Absolute number × 10^6^/L. Peripheral blood mononuclear cells (PBMC) were identified and quantified after surface staining with monoclonal antibodies specific for anti-human CD45^+^, CD3^+^, CD4^+^, CD25^hi^, CD8^+^, CD56^+^, CD19^+^, CD14^+^, HLA-DR, TLR4. Phenotypic analysis of white blood cells were determined by 10-color flow cytometry: CD45^+^, General Leukocyte marker; CD3^+^, T-cell marker; CD3^+^ CD4^+^, CD4^+^ T-helper marker; CD3^+^ CD4^+^ CD25^hir^, T-regulator cell marker; CD3^+^ CD8^+^, Cytotoxic T-cell marker; CD3^–^ CD56^+^, Natural Killer cell marker; CD19^+^, B-cell marker; CD14^+^, monocyte marker; HLA-DR, activation marker for T-cells and monocytes; CD25, activation marker for T-cells; TLR4, Toll-like receptor 4 marker; CD3^+^ CD4^–^ CD8^–^, double negative T-cell (DNT); CD3^+^ CD4^+^ CD8^+^, double positive T-cell (DPT). ^†^ANOVA between groups. ^‡^Post hoc comparison healthy participants versus cervical radiculopathy. ^#^Post hoc comparison non-specific neck pain versus cervical radiculopathy. ^§^ Post hoc comparison non-specific neck pain versus healthy participants. Significant differences between groups are in bold (p < 0.05).

#### Inflammatory marker concentration following *in vitro* stimulation of whole blood cells

**FIGURE 3:**
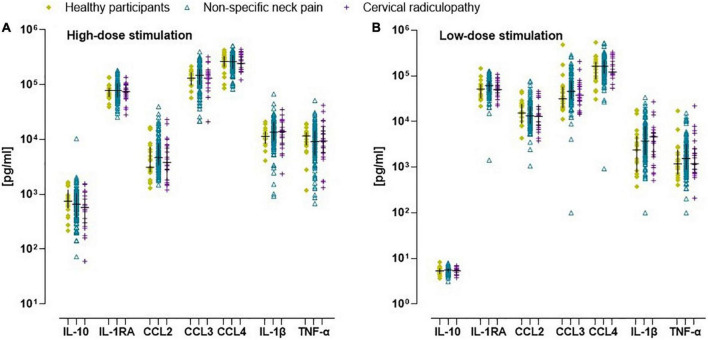
Scatter plot and median (inter quartile range) of *in vitro* concentration of inflammatory markers in healthy participants, people with non-specific neck pain, and cervical radiculopathy. **(A)** Crude *in vitro* inflammatory marker concentration after whole blood stimulation with TLR4 agonist lipopolysaccharide at a concentration of 10 μg/ml (high dose-LPS stimulation) in healthy participants, people with non-specific neck pain, and cervical radiculopathy. **(B)** The raw *in vitro* inflammatory marker concentration after whole blood stimulation with TLR4 agonist lipopolysaccharide at a concentration of 1 ng/ml (low dose-LPS stimulation) in healthy participants, people with non-specific neck pain, and cervical radiculopathy. To determine significant differences between groups the *in vitro* values were first Ln-transformed and finally expressed/1,000 monocytes. Lines represent median (*horizontal line*), and 25th–75th percentiles. TNF-α, tumor necrosis factor-α; IL-1β, interleukin-1β; IL-1RA, interleukin-receptor antagonist; IL-4, interleukin-4; IL-10, interleukin-10; CCL2, c-c-motif ligand 2 also referred to as monocyte chemoattractant protein 1; CCL3, c-c-motif ligand 3 also referred to as macrophage inflammatory protein 1α; CCL4, c-c-motif ligand 4 also referred to as macrophage inflammatory protein 1β. The scatter plot was based on the individual points of 112 people with non-specific neck pain, 25 with cervical radiculopathy and 23 healthy participants.

All inflammatory markers were Ln-transformed to normalize the residual variances. In the non-specific neck pain group, the crude and monocyte normalized models revealed no significant difference for the inflammatory indices and the single inflammatory markers (Figures 1B–D). For the people with a cervical radiculopathy, low-dose whole blood stimulation revealed reduced inflammatory (β = −0.11), pro-inflammatory (β = −0.10), and anti-inflammatory (β = −0.15) indices in the crude model, which disappeared in the monocyte normalized model (Figures 1B–D and Supplementary material E). There were no differences between people with non-specific neck pain and cervical radiculopathy. Sex was an effect modifier in the non-specific neck pain and cervical radiculopathy group (Figure 3 and Supplementary material E).

### Associations between inflammatory indices and clinical, psychological, and lifestyle factors

**FIGURE 4:**
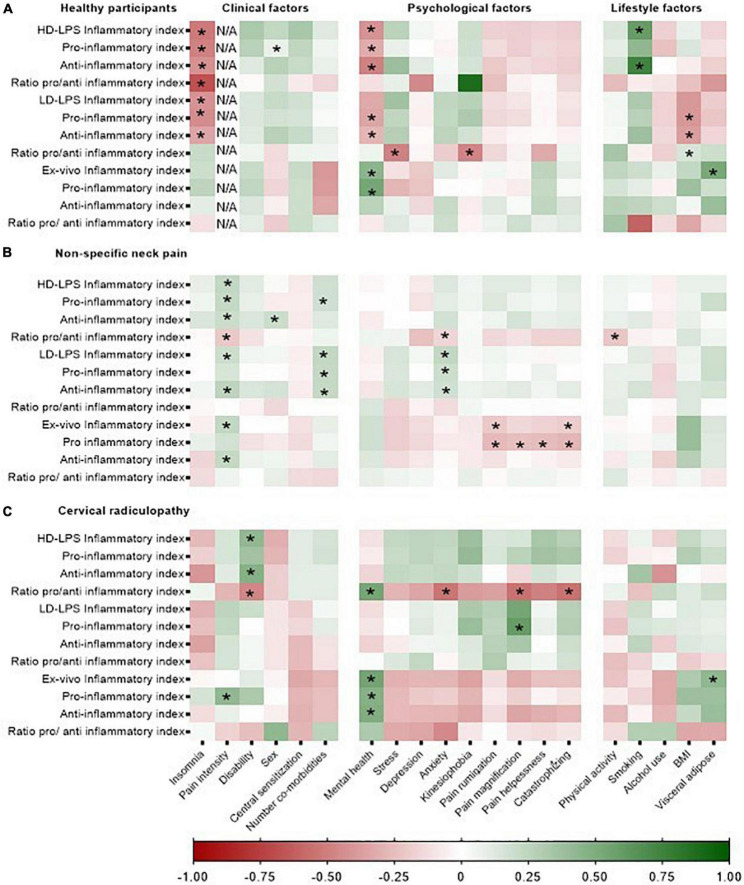
Heatmap representing the strength of the associations between clinical, psychological, and lifestyle factors and the *in vitro* whole blood response and inflammatory indices in healthy participants, non-specific neck pain, and cervical radiculopathy. Inflammatory markers were Ln-transformed and expressed as standardized-B. *In vitro* inflammatory indices were /1,000 monocytes normalized. HD-LPS, high dose – LPS stimulation, 10 μg/ml; LD-LPS, low-dose LPS stimulation, 1 ng/ml. “*”Represent a significant association (*p* < 0.05). The heatmap was based on the data of 112 people with non-specific neck pain, 25 with cervical radiculopathy, and 23 healthy participants. **(A)** Represents the heatmap for the healthy participants, **(B)** represents the heatmap for the people with non-specific neck pain and **(C)** represents the heatmap of the people with a cervical radiculopathy.

In all groups, several clinical, psychological and lifestyle factors were associated with the *ex vivo* concentrations of inflammatory indices and *in vitro* whole blood responsiveness (Figure 4). For the people with non-specific neck pain, the standardized coefficient-β varied between β = −0.21 (pain intensity) and β = 0.25 (pain intensity) for the clinical factors, between β = −0.28 (catastrophizing) and β = 0.23 (anxiety symptoms) for the psychological factors and between β = −0.22 (physical activity) and β = 0.39 (BMI) for the lifestyle factors (Supplementary material F). For people with cervical radiculopathy, the association between neuroimmune responses and clinical, psychological, and life style factors varied between β = −0.47 (disability) and β = 0.42 (pain intensity) for the clinical factors, between β = −0.46 (anxiety) and β = 0.59 (pain magnification) for the psychological factors and β = 0.43 (visceral adipose tissue) for the lifestyle factors (Supplementary material G). The healthy participants showed associations between β = −0.68 (number co-morbidities) and β = 0.47 (sex) for the clinical factors, between β = −0.49 (mental health) and β = 0.53 (mental health) for the psychological factors and between β = −0.61 (smoking) and β = 0.74 (smoking) for the lifestyle factors (Supplementary material H). Additionally, the association between the single inflammatory markers and clinical, psychological and lifestyle factors for females and males can be retrieved from Supplementary material F–H.

## Discussion

This study was designed to evaluate a broad range of systemic neuroimmune responses in people with non-specific neck pain, people with a cervical radiculopathy and healthy participants, and to explore the associations between neuroimmune responses with clinical, psychological and lifestyle factors. The major findings of this study were the upregulated serum *ex vivo* inflammatory indices in both the non-specific neck pain group and cervical radiculopathy group compared to the healthy participants, and the significant associations between the inflammatory indices and clinical, psychological and lifestyle factors, such as pain intensity, disability, and mental health. We did not detect meaningful differences in neuroimmune responses after *in vitro* whole blood stimulation between the three groups.

### Systemic neuroimmune responses

Approximately one in three people with non-specific neck pain and one in five people with a cervical radiculopathy showed CRP levels >3 mg/L, a widely used threshold to define low-grade inflammation (Pearson et al., 2003). Inflammation levels in people with non-specific neck pain seem to be important, particularly as a state of low-grade systemic inflammation is associated with poor recovery and other highly prevalent co-morbid conditions, such as depression and cardiovascular disease (Sterling et al., 2013; Koop et al., 2021). Systemic inflammation could be a shared mechanism for these conditions. Despite the upregulated *ex vivo* concentration of inflammatory markers, we did not detect any upregulated *in vitro* response of whole blood cells to LPS.

The technique of *in vitro* evoked-release of inflammatory markers is often performed and enhanced inflammatory markers in different persistent pain conditions have frequently been found (Teodorczyk-Injeyan et al., 2011, 2015, 2019; Kwok et al., 2012). However, we were unable to find increased *in vitro* responsivity of peripheral immunocompetent cells in people with non-specific neck pain and cervical radiculopathy compared to healthy participants. The discrepancy between our findings regarding the *in vitro* stimulation of immunocompetent cells and other literature are unlikely to be explained by differences in methodology. To date, laboratories have employed diverse techniques to determine and report *in vitro* responsivity of peripheral immunocompetent cells in patients with persistent pain, complicating interpretation, comparisons and reproducibility (Segre and Fullerton, 2016). Methodological heterogeneity, such as source and concentration of LPS, incubation time, predilution, whole-blood cultures versus stimulation of PBMC, can affect the production of cytokines. In our study, time between blood withdrawal and whole blood stimulation was set at 4-h leading to lower release compared to immediate stimulation. However, as the experimental methods were identical between the three groups, we controlled for these factors (Yaqoob et al., 1999; Damsgaard et al., 2009; Segre and Fullerton, 2016).

A strength of our research was that we were able to test for differences in the number of cytokine-producing cells in whole blood (Yaqoob et al., 1999; Candore et al., 2006; Segre and Fullerton, 2016). Controlling for whole blood monocyte count did however not change our findings. Using FACS analysis, we were unable to find differences in activation status (HLA-DR^+^ monocytes/CD14^+^) and TLR4 expression on monocytes of the cultured cells between groups. This finding is supported by a study which did not find systemic differences in pro-inflammatory (CD14^+^ CD16^+^) monocytes in patients with non-specific neck pain compared to healthy participants (Li et al., 2016). Next to monocytes, neutrophils are also TLR4^+^ and responsive to LPS stimulation, and therefor able to induce the production of cytokines (Sabroe et al., 2005). However, as we did not measure neutrophil levels we were unable to control for neutrophil counts. Another research group also failed to identify increased responsivity of immunocompetent cells after TLR4 stimulation *in vitro* in chronic neuropathic pain conditions (Langjahr et al., 2018). We took care to exclude patients with inflammatory co-morbidities, certain medications and clinical psychological conditions, as these have all been associated with increased neuroimmune responses, to ensure our findings are related to the pain state (Segre and Fullerton, 2016; Rogero and Calder, 2018; Wang et al., 2020; Koop et al., 2021). Certain comorbidities (e.g., high blood pressure and cardiac arrhythmia) and musculoskeletal pain at other locations were not excluded in order to be able to recruit sufficient participants. We believe this was justified as neck pain and neck-arm pain were the dominant pain sites, and the condition for which treatment was sought. The exclusion of clinical psychological conditions might in part explain our negative findings on *in vitro* whole blood responsivity. Previous research in people with multisite chronic pain revealed that the association between the inflammatory index and persistent pain became non-significant following adjustment for depression and anxiety (Generaal et al., 2014).

A consideration has to be made regarding sample sizes. The control group consisted of 23 participants despite sample size calculation suggesting at least 25 per group. However, as 15% sample failure for TNF-α was taken into account in the sample size estimation, our sample size of 23 in the control group was sufficient. The larger than necessary inclusion of people with non-specific neck pain resulted in a more precise estimate of the neuroimmune responses.

### Associations between systemic neuroimmune responses and clinical, psychological, and lifestyle factors

We found significant associations between cytokine production and clinical, psychological and lifestyle factors with significant effect modification by sex. Among multiple other significant associations, the most frequent were between the *in vitro* inflammatory indices and pain intensity for people with non-specific neck pain, disability for cervical radiculopathy, and insomnia for the healthy participants (Figure 4). There are several potential pathways how circulating inflammatory markers might affect pain and nervous system signaling. The nociceptor terminals and dorsal root ganglia are directly exposed to circulating products which may support sensitization and excitation (Gonçalves Dos Santos et al., 2019). In addition, systemic cytokine signals are able to reach the spinal cord and supraspinal regions of the pain neuraxis through humoral (e.g., leaky regions of the blood-brain barrier), neural (e.g., transmission *via* afferent nerve fibers) and cellular pathways (e.g., recruitment of monocytes into the brain) (Capuron and Miller, 2011) affecting pain and sickness behavior. However, as we used a cross-sectional design, cause-effect associations are not possible (Lutke Schipholt et al., 2018). It would be informative to examine the time sequences between the neuroimmune responses in relation to the different clinical, psychological, and lifestyle factors, and to establish the therapeutic potential of these responses.

There are several noteworthy findings when scrutinizing the association between the neuroimmune responses and the various risk factors for persistent pain. While the evidence is overwhelming that physical activity has immune regulatory effects (Plaisance and Grandjean, 2006), we did not find an association between physical activity levels and inflammatory indices. One likely explanation might be that participants may not accurately complete physical activity questionnaires. Wearable activity trackers may be more valid to document physical activity (van Poppel et al., 2010; Chastin et al., 2014; Chan et al., 2022). Another remarkable association was the inverse association between catastrophizing with several neuroimmune responses. It is well known that an upregulated neuroimmune system might induce sickness behavior and there is abundant evidence of positive associations between inflammatory neuroimmune responses and clinical psychological conditions (Wium-Andersen et al., 2013). However, several studies have also found inverse or no associations between neuroimmune responses and psychological factors (Dowlati et al., 2010; Lehto et al., 2010; Vogelzangs et al., 2013; Yirmiya et al., 2015). These discrepancies in the literature might be explained as psychological stressors may induce immunosuppression by for example stimulation of the hypothalamic-pituitary-adrenal and sympathetic-adrenal-medullary-axis (Elenkov et al., 2000; Segerstrom and Miller, 2004; Yirmiya et al., 2015).

To conclude, as the *ex vivo* pro-inflammatory indices were enhanced and associated with pain intensity, further prospective and interventional research is warranted to provide insight in the cause-effect relationship and the therapeutic potential of reducing the systemic pro inflammation in people with non-specific neck pain and radiculopathy.

## Data availability statement

The raw data supporting the conclusions of this article will be made available by the authors, without undue reservation.

## Ethics statement

The studies involving human participants were reviewed and approved by the Medical Ethics Committee of Amsterdam University Medical Centre, location VUmc (Approval number: 2018.181) and was registered at trialregister.nl (study ID: NL6575). The patients/participants provided their written informed consent to participate in this study.

## Author contributions

IL: conceptualization, methodology, data curation, formal analysis, writing—original draft preparation, project administration, and funding acquisition. GS-P and MC: conceptualization, methodology, data curation, formal analysis, writing—review and editing, supervision, and funding acquisition. MK and PB: investigation, resources, and writing—review and editing. HB: conceptualization, methodology, investigation, resources, and writing—review and editing. All authors contributed to the article and approved the submitted version.
