# Supplementary material for: Systemic neuroimmune responses in people with non-specific neck pain and cervical radiculopathy, and associations with clinical, psychological, and lifestyle factors
Source: Front Mol Neurosci. 2022 Oct 13;15:1003821. doi: 10.3389/fnmol.2022.1003821 (PMC9608367; doi:10.3389/fnmol.2022.1003821)
Supplement: Supplementary file 1 [file Data_Sheet_1.docx]

**SUPPLEMENTARY A** Gating strategy for flow cytometry of white blood cells

**
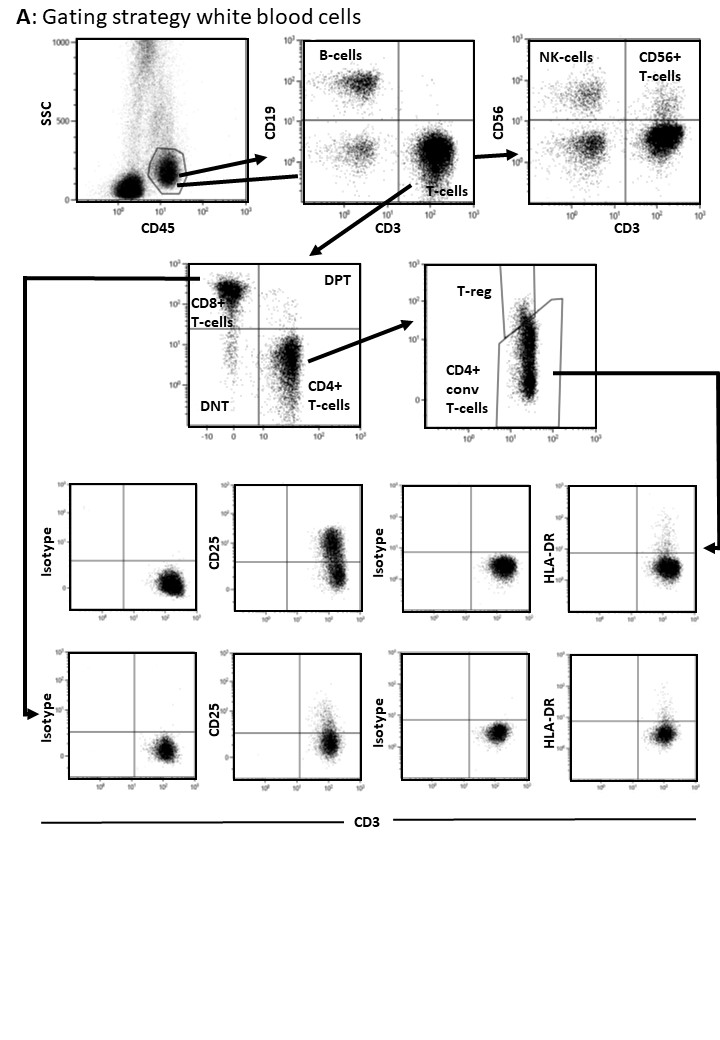

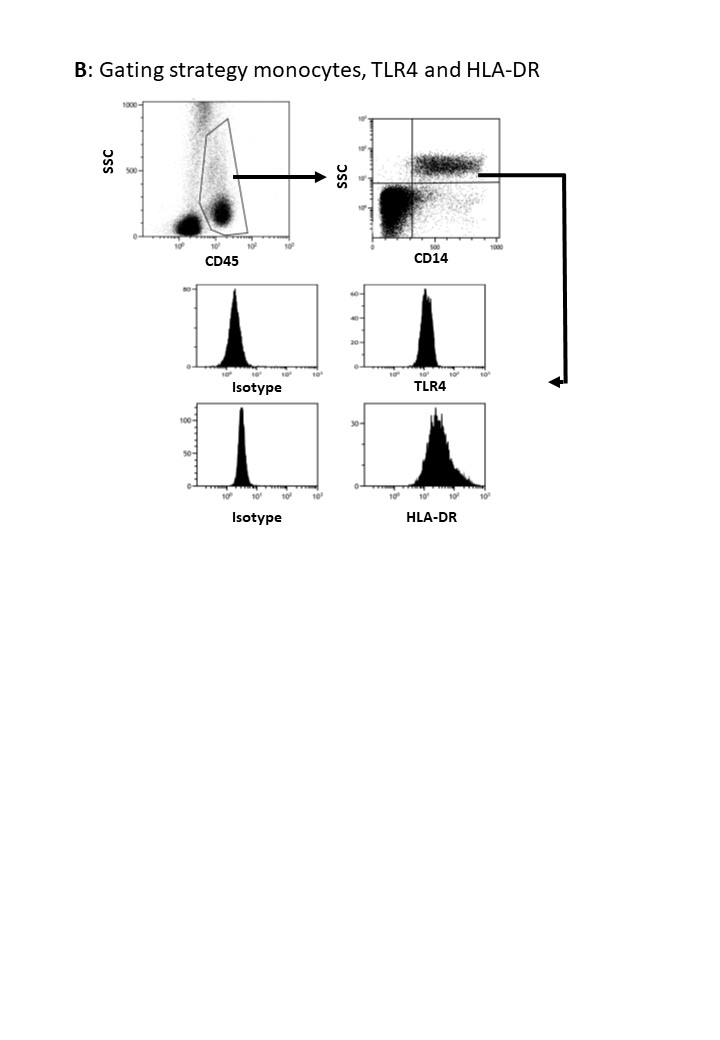
**

Panel **A:** Lymphocytes are gated based on low side scatter (SSC) and CD45 expression. Within the lymphocytes, CD19^+^ B-cells, CD3^+^ T-cells, CD56^+^CD3^-^ natural killer (NK) cells and CD56^+^ T-cells are gated. Within CD3^+^ T-cells CD4^+^CD8^-^ (CD4^+^ T-cells), CD4^-^CD8^+^ (CD8^+^ T-cells), CD4^+^CD8^+^ double positive T-cells (DPT) and CD4^-^ D8^-^ double negative T-cells (DNT) are gated. Within CD4^+^ T-cells, CD25hi T-regulatory (Treg) are gated, the remaining CD4^+^ T-cells are defined as conventional T-cells. CD25 and HLA-DR expression is analyzed on CD4^+^ conventional T-cells and CD8^+^ T-cells using appropriate isotype controls for gating. Panel **B:** Mononuclear cells (PBMC) are gated based on low/intermediate SSC and CD45 expression. Within the PBMC, CD14^+^ monocytes are gated. Activation markers TLR4 and HLA-DR expression on CD14^+^ monocytes is analysed and expression levels are corrected for background staining with appropriate isotype controls.

**SUPPLEMENTARY B**

| **Clinical factors** | **Psychological factors** | **Lifestyle factors** |
| --- | --- | --- |
| Pain intensity (Matute Wilander, Kåredal et al. 2014) | Mental health (Wium-Andersen, Ørsted et al. 2013) | Physical activity (Abramson and Vaccarino 2002) |
| Disability (Klyne, Barbe et al. 2022) | Stress (Segerstrom and Miller 2004) | Smoking (Shiels, Katki et al. 2014) |
| Sex (Gregus, Levine et al. 2021) | Depression (Wium-Andersen, Ørsted et al. 2013) | Alcohol use (Klyne, Barbe et al. 2017) |
| Central sensitization (Fitzcharles, Cohen et al. 2021) | Anxiety (Vogelzangs, Beekman et al. 2013) | Insomnia (Klyne, Barbe et al. 2017) |
| Number of co-morbidities (Koop, Lutke Schipholt et al. 2021) | Kinesiophobia (Koop, Lutke Schipholt et al. 2021) | BMI (Klyne, Barbe et al. 2017) |
|  | Rumination (Klyne, Barbe et al. 2017) | Visceral adipose tissue (Schlecht, Fischer et al. 2016) |
|  | Magnification (Klyne, Barbe et al. 2017) |  |
|  | Helplessness (Klyne, Barbe et al. 2017) |  |
|  | Catastrophizing (Klyne, Barbe et al. 2017) |  |

**SUPPLEMENTARY C**  Flowchart of the study

**SUPPLEMENTARY D** Differences in ex-vivo inflammatory marker concentration between healthy participants, people with non-specific neck pain and cervical radiculopathy.

|  | **Non-specific neck pain versus healthy participants** | **Cervical radiculopathy versus healthy participants** | **Non-specific neck pain versus cervical radiculopathy** |
| --- | --- | --- | --- |
| Ex-vivo | B^1^ (SE) | B^1^ (SE) | B^1^ (SE) |
| Inflammatory index | **0.54 (0.24)** | 0.55 (0.28)* | 0.16 (0.25) |
| Pro-inflammatory index | **0.70 (0.24)** | **0.64 (0.30)** | 0.23 (0.25) |
| Anti-inflammatory index | 0.39 (0.31) | 0.47 (0.31) | 0.09 (0.33) |
| Ratio pro/anti inflammatory index | 8.16 (4.77) | 12.05 (8.30) | -1.16 (3.65) |
| hsCRP | 0.52 (0.29) | 0.59 (0.30)* | 0.27 (0.30) |
| IL-1β | **1.85 (0.57)** | 2.42 (2.49) | 1.33 (0.93) |
| IL-1RA | **0.33 (0.12)** | **0.35 (0.14)** | 0.008 (0.14) |
| TNF-α | **0.36 (0.14)** | 0.29 (0.17) | 0.07 (0.16) |
| sTNF-R2 | -0.03 (0.09) | -0.003 (0.04) | 0.08 (0.11) |

Table shows the differences in *ex-vivo* inflammatory indexes and inflammatory markers in healthy participants, people with non-specific neck pain and cervical radiculopathy. The *ex-vivo* values were Ln-transformed. The inflammatory index was calculated as the sum of z-standardized hsCRP, IL-1β, TNF-α, IL-1RA and sTNF-R2 divided by the number of cytokines. Pro-inflammatory index was calculated as the sum of z-standardized hsCRP, IL-1β and TNF-α. Anti-inflammatory index was calculated as the sum of z-standardized IL-1RA and STNF-R2. TNF-α, tumor necrosis factor -α; IL-1β, interleukin -1β; IL-1RA, interleukin -receptor antagonist; Bold = P < 0.05; * = P = 0.06; ^1^Ln-transformed, adjusted for plate number

**SUPPLEMENTARY E**

Differences in inflammatory marker concentration after *in-vitro* stimulation of whole blood cells in healthy participants, people with non-specific neck pain and cervical radiculopathy.

|  |  | **Non-specific neck pain versus healthy participants** | **Non-specific neck pain versus healthy participants** | **Cervical radiculopathy versus healthy participants** | **Cervical radiculopathy versus healthy participants** | **Non-specific neck pain versus cervical radiculopathy** | **Non-specific neck pain versus cervical radiculopathy** |
| --- | --- | --- | --- | --- | --- | --- | --- |
| In-vitro | LPS | B^1^ (SE) | Normalized-B^2^ (SE) | B^1^ (SE) | Normalized-B^2^ (SE) | B^1^ (SE) | Normalized-B^2^ (SE) |
| Inflammatory index | LD-LPS | -0.19 (0.20) | -0.11 (0.21) | **-0.11 (0.04)** | -0.08 (0.05) | -0.07 (0.18) | -0.05 (0.19) |
|  | HD-LPS | -0.10 (0.22)  ♂ -0.21 (0.28)  ♀ 0.31 (0.33) | 0.004 (0.26) | -0.09 (0.06)  ♂ -0.32 (0.30)  ♀ 0.22 (0.28) | -0.02 (0.06) | 0.08 (0.21) | 0.10 (0.24) |
| Pro-inflammatory index | LD-LPS | -0.17 (0.24)  ♂ -0.36 (0.19)  ♀ 0.42 (0.48) | -0.10 (0.21) | **-0.10 (0.04)** | -0.06 (0.05) | -0.09 (0.19) | -0.07 (0.19) |
|  | HD-LPS | -0.04 (0.18) | 0.06 (0.26) | -0.11 (0.06)  ♂ -0.04 (0.33)  ♀ 0.28 (0.33) | -0.04 (0.06) | -0.005 (0.23) | 0.007 (0.25) |
| Anti-inflammatory index | LD-LPS | -0.24 (0.21)  ♂ -0.40 (0.24)  ♀ -0.09 (0.47) | -0.14 (0.23) | **-0.15 (0.05)** | -0.11 (0.06) | -0.003 (0.20) | 0.007 (0.21) |
|  | HD-LPS | -0.27 (0.26) | -0.13 (0.31) | -0.04 (0.07) | -0.02 (0.08) | 0.30 (0.24) | 0.33 (0.29) |
| Ratio pro/anti inflammatory index | LD-LPS | -0.86 (1.93) | -0.21 (0.75) | -0.04 (0.27) | -0.22 (1.01) | -1.82 (1.79) | -3.61 (1.89) |
|  | HD-LPS | 7.88 (27.7) | -1.09 (3.93) | -0.29 (0.21) | -0.13 (0.25) | 9.80 (25.0) | -0.03 (3.54) |
| TNF-α | LD-LPS | -0.37 (0.23) | -0.31 (0.24) | -0.04 (0.27) | 0.02 (0.31) | -0.31 (0.22) | -0.30 (0.23) |
|  | HD-LPS | -0.23 (0.18)  ♂ -0.27 (0.22)  **♀** 0.16 (0.30) | -0.17 (0.20) | -0.11 (0.25) | -0.05 (0.25) | -0.15 (0.18) | -0.14 (0.19) |
| IL-1β | LD-LPS | -0.04 (0.26) | 0.02 (0.29) | 0.34 (0.32) | 0.40 (0.38) | -0.32 (0.29) | -0.31 (0.26) |
|  | HD-LPS | 0.12 (0.16) | 0.18 (0.19) | 0.17 (0.16) | 0.22 (0.20) | -0.001 (0.15) | 0.005 (0.18) |
| IL-1RA | LD-LPS | -0.07 (0.14)  ♂ -0.13 (0.14)  ♀ 0.27 (0.35) | -0.12 (0.16)  ♂ -0.36 (0.20)  ♀ -0.06 (0.16) | -0.03 (0.13) | 0.03 (0.16) | -0.005 (0.13) | 0.002 (0.15) |
|  | HD-LPS | -0.08 (0.10) | -0.02 (0.12)  ♂ -0.02 (0.16)  ♀ -0.20 (0.21) | -0.04 (0.11) | 0.02 (0.12) | -0.006 (0.10) | 0.001 (0.12) |
| IL-4 | LD-LPS | - | - | - | - | - | - |
|  | HD-LPS | - | - | - | - | - | - |
| IL-10 | LD-LPS | -0.33 (0.21) | -0.28 (0.22) | -0.21 (0.21) | -0.15 (0.25) | 0.004 (0.20) | 0.01 (0.21) |
|  | HD-LPS | -0.16 (0.19) | -0.10 (0.19) | -0.42 (0.23) | -0.36 (0.23) | 0.35 (0.18) | 0.35 (0.18) |
| CCL2 | LD-LPS | 0.03 (0.18)  ♂ 0.13 (0.35)  ♀ 0.008 (0.20) | 0.08 (0.18) | -0.23 (0.23)  ♂ -0.19 (0.30)  ♀ -0.09 (0.26) | -0.17 (0.21) | 0.25 (0.17) | 0.25 (0.17) |
|  | HD-LPS | 0.15 (0.18)  ♂ 0.11 (0.21)  ♀ 0.31 (0.34) | 0.20 (0.19) | -0.03 (0.24) | 0.03 (0.26) | 0.16 (0.17) | 0.17 (0.18) |
| CCL3 | LD-LPS | -0.20 (0.24) | -0.14 (0.26) | 0.02 (0.19) | 0.07 (0.24) | -0.13 (0.22) | -0.13 (0.23) |
|  | HD-LPS | -0.04 (0.14)  ♂ -0.13 (0.18)  ♀ 0.31 (0.23) | 0.02 (0.17) | -0.001 (0.15) | 0.06 (0.16) | 0.01 (0.14) | 0.02 (0.15) |
| CCL4 | LD-LPS | -0.18 (0.19) | -0.12 (0.20) | -0.06 (0.14)  ♂ -0.15 (0.20)  ♀ 0.09 (0.17) | -0.005 (0.18) | -0.08 (0.17) | -0.07 (0.19) |
|  | HD-LPS | -0.10 (0.10)  ♂ -0.14 (0.13)  ♀ 0.06 (0.15) | -0.04 (0.13) | -0.04 (0.11) | 0.02 (0.13) | -0.02 (0.10) | -0.01 (0.12) |

Table shows the differences in inflammatory indices and inflammatory markers after whole blood stimulation with TLR4 agonist lipopolysaccharide at concentration of 1ng/ml (LD-LPS) or 10µg/ml (HD-LPS) in healthy participants, people with non-specific neck pain and cervical radiculopathy. The inflammatory index was calculated as the sum of z-standardized (/1000 monocyte normalized) CCL2, CCL3, CCL4, TNF-α, IL-1β, IL-10 and IL-1RA divided by the number of cytokines. Pro-inflammatory index was calculated as the sum of z-standardized (/1000 monocyte normalized) CCL2, CCL3, CCL4, TNF-α and Il-1β. Anti-inflammatory index was calculated as the sum of z-standardized (/1000 monocyte normalized) IL-1RA and IL-10. TNF-α, tumor necrosis factor -α; IL-1β, interleukin -1β; IL-1RA, interleukin -receptor antagonist; IL-4, interleukin – 4; IL-10, interleukin -10; CCL2, c-c-motif ligand 2 also referred to as monocyte chemoattractant protein 1; CCL3, c-c-motif ligand 3 also referred to as macrophage inflammatory protein 1α; CCL4, c-c-motif ligand 4 also referred to as macrophage inflammatory protein 1β; LPS, lipopolysaccharide; Bold = p < 0.05. ♂ effect modification for males; ♀ effect modification for females.

^1^Ln-transformed, adjusted for plate number, LPS lot number, stimulation time

^2^Ln-transformed, normalized (/1000 monocytes), adjusted for plate number, LPS lot number, stimulation time.

**SUPPLEMENTARY F1** Association of lifestyle factors with neuroimmune responses in people with non-specific neck pain.

|  | | **Physical Activity^a^** | **Smoking^b^** | **Alcohol use^c^** | **BMI** | **Visceral Adipose^d^** |
| --- | --- | --- | --- | --- | --- | --- |
|  | | **Standardized- adjusted** β **(SE)** | **Standardized- adjusted** β **(SE)** | **Standardized- adjusted** β **(SE)** | **Standardized- adjusted** β **(SE)** | **Standardized- adjusted** β **(SE)** |
| **In-vitro** | | | | | | |
| Inflammatory index | LD-LPS | 0.20 | 0.11 (0.18) | -0.11 (0.15) | 0.07 (0.02) | **0.19 (0.003)** |
|  | HD-LPS | 0.06 (0.007) | 0.04 (0.16) | -0.06 (0.14) | 0.08 (0.02) | 0.08 (0.003) |
| Pro-inflammatory index | LD-LPS | 0.08 (0.008) | 0.08 (0.18) | -0.16 (0.16) | 0.05 (0.02) | **0.20 (0.003)** |
|  | HD-LPS | 0.05 (0.007) | 0.002 (0.17) | -0.05 (0.14) | 0.07 (0.02) | 0.10 (0.003) |
| Anti-inflammatory index | LD-LPS | 0.09 (0.008) | 0.16 (0.19) | -0.15 (0.17) | 0.13 (0.02) | 0.15 (0.004) |
|  | HD-LPS | 0.08 (0.008) | 0.11 (0.18) | -0.07 (0.15) | 0.09 (0.02) | 0.02 (0.003) |
| Ratio pro/anti inflammatory index | LD-LPS | 0.10 (0.07) | 0.001 (1.60) | -0.03 (1.37) | 0.13 (0.15)  ♂ -0.18 (0.17)  ♀ 0.22 (0.20) | 0.07 (0.01) |
|  | HD-LPS | **-0.22 (0.08)** | -0.05 (1.72) | -0.03 (1.46) | 0.14 (0.16) | 0.06 (0.03) |
| TNF-α | LD-LPS | 0.06 (0.009) | 0.002 (0.21) | -0.06 (0.18) | -0.04 (0.003) | **0.25 (0.004)** |
|  | HD-LPS | 0.06 (0.008) | -0.07 (0.17) | 0.02 (0.15) | -0.05 (0.002) | 0.04 (0.003) |
| IL-1β | LD-LPS | 0.05 (0.01) | 0.15 (0.24) | -0.16 (0.20) | -0.06 (0.003) | 0.14 (0.004) |
|  | HD-LPS | 0.04 (0.007) | -0.03 (0.17) | -0.10 (0.14) | -0.02 (0.002) | 0.05 (0.003) |
| IL-1RA | LD-LPS | 0.11 (0.006) | 0.25 (0.14) | **-0.19 (0.12)**  ♂ -0.26 (0.32)  ♀ -0.01 (0.11) | 0.04 (0.002) | **0.22 (0.003)**  ♂ 0.32 (0.006)  ♀ 0.27 (0.002) |
|  | HD-LPS | 0.15 (0.005)  ♂ 0.21 (0.007)  **♀ 0.17 (0.006)** | 0.17 (0.11) | -0.15 (0.09) | 0.005 (0.002)  ♂ 0.44 (0.02)  ♀ 0.17 (0.01) | 0.14 (0.002)  ♂ 0.32 (0.003)  ♀ 0.17 (0.002) |
| IL-4 | LD-LPS | - | - | - | - | - |
|  | HD-LPS | **-** | **-** | **-** | **-** | **-** |
| IL-10 | LD-LPS | 0.05 (0.008) | 0.13 (0.19) | -0.06 (0.16) | -0.16 (0.003) | 0.03 (0.003) |
|  | HD-LPS | -0.02 (0.007) | 0.006 (0.16) | 0.04 (0.07) | -0.13 (0.002) | -0.11 (0.003) |
| CCL2 | LD-LPS | 0.05 (0.007) | 0.07 (0.16) | -0.02 (0.12) | -0.04 (0.002) | 0.02 (0.003) |
|  | HD-LPS | -0.06 (0.007) | 0.09 (0.16) | -0.13 (0.13) | -0.06 (0.002) | 0.08 (0.003) |
| CCL3 | LD-LPS | 0.06 (0.010) | 0.04 (0.22) | -0.09 (0.19) | -0.04 (0.003) | **0.20 (0.004)** |
|  | HD-LPS | 0.03 (0.006) | -0.05 (0.15) | 0.021 (0.12) | -0.06 (0.002) | 0.08 (0.003) |
| CCL4 | LD-LPS | 0.09 (0.008) | 0.05 (0.18) | -0.07 (0.15) | -0.04 (0.002) | **0.20 (0.003)** |
|  | HD-LPS | 0.11 (0.005) | 0.06 (0.12) | -0.004 (0.10) | -0.06 (0.002) | 0.14 (0.002) |
| **Ex-vivo** | | | | | | |
| Inflammatory index |  | -0.03 (0.007) | 0.01 (0.14) | -0.08 (0.13) | **0.39 (0.01)** | 0.14 (0.003) |
| Pro-inflammatory index |  | 0.04 (0.007) | -0.03 (0.15) | 0.05 (0.15) | **0.39 (0.02)** | 0.12 (0.003) |
| Anti-inflammatory index |  | -0.08 (0.008) | 0.05 (0.18) | -0.17 (0.16) | **0.28 (0.02)** | 0.12 (0.003) |
| Ratio pro/ anti inflammatory index |  | 0.12 (0.29) | -0.03 (5.96) | -0.07 (5.56) | 0.04 (0.61) | -0.09 (0.12) |
| hsCRP |  | 0.01 (0.01) | 0.05 (0.26) | -0.01 (0.25) | **0.44 (0.02)** | 0.16 (0.005) |
| TNF-α |  | 0.07 (0.006) | -0.09 (0.13) | 0.08 (0.12) | 0.12 (0.01)  ♂ 0.30 (0.02)  ♀ 0.006 (0;01) | 0.03 (0.002)  **♂ 0.14 (0.005)**  ♀-0.12 (0.003) |
| sTNF-R2 |  | -0.10 (0.004) | 0.06 (0.08) | -0.13 (0.08) | 0.07 (0.009) | 0.05 (0.002) |
| Il-1β |  | 0.03 (0.03) | 0.12 (0.56) | 0.002 (0.49) | 0.22 (0.05) | 0.16 (0.01) |
| IL-1RA |  | -0.03 (0.005) | 0.02 (0.11) | -0.15 (0.10) | **0.40 (0.01)** | 0.14 (0.002) |

Data represent standardized regression coefficient β (standard error, unstandardized B) of various lifestyle factors demonstrating an association with *ex-vivo* and *in-vitro* inflammatory indexes and of *in-vitro* and *ex-vivo* single inflammatory mediators using linear regression analysis. All single neuroimmune responses are ln-transformed. The *in-vitro* neuroimmune responses are normalized for monocyte count. Significant values are in bold font (^*^p < 0.05). TNF-α, tumor necrosis factor -α; IL-1β, interleukin -1β; IL-1RA, interleukin -receptor antagonist; IL-4, interleukin – 4; IL-10, interleukin -10; CCL2 / MCP1, c-c-motif ligand 2 also referred to as monocyte chemoattractant protein 1; CCL3 / MIP1α, c-c-motif ligand 3 also referred to as macrophage inflammatory protein 1α; CCL4 / MIP1β, c-c-motif ligand 4 also referred to as macrophage inflammatory protein 1β; TNFα : IL-10, ratio between TNFα : IL-10; IL-1β : IL-1RA, ratio between IL-1β : IL-1RA; 1ng/ml (LD-LPS), 1 milliliter whole-blood stimulation with 1 nanogram TLR4 agonist lipopolysacharide; 10µg/ml (HD-LPS), 1 milliliter whole blood stimulation with 10 microgram of TLR4 agonist lipopolysacharide; ♂ effect modification for males; ♀ effect modification for females.

^a^Refers to 1000/metabolic equivalent of a task

^b^Refers to current smoker, yes:1, no:0

^c^Refers to consuming alcohol, yes:1, no:0

^d^Refers to linear distance in millimeters between the posterior aspect of peritoneum and anterior aspect of lumbar vertebra T10

**SUPPLEMENTARY F2** Association of clinical factors with neuroimmune responses in people with non-specific neck pain.

|  | | **Pain intensity^a^** | **Disability^b^** | **Sex^c^** | **Central Sensitization^d^** | **Number of co-morbidities** | **Insomnia^e^** |
| --- | --- | --- | --- | --- | --- | --- | --- |
|  | | **Standardized- adjusted** β **(SE)** | **Standardized- adjusted** β **(SE)** | **Standardized- adjusted** β **(SE)** | **Standardized- adjusted** β **(SE)** | **Standardized- adjusted** β **(SE)** | **Standardized- adjusted** β **(SE)** |
| **In-vitro** | | | | | | |  |
| Inflammatory index | LD-LPS | **0.19 (0.004)** | 0.05 (0.006) | 0.04 (0.16) | -0.06 (0.03) | **0.23 (0.06)** | 0.008 (0.17) |
|  | HD-LPS | **0.23 (0.003)** | 0.11 (0.005) | 0.02 (0.14) | -0.08 (0.03) | 0.18 (0.05) | 0.09 (0.15) |
| Pro-inflammatory index | LD-LPS | 0.15 (0.004) | 0.009 (0.006) | -0.01 (0.16) | -0.05 (0.03) | **0.22 (0.06)** | -0.01 (0.17) |
|  | HD-LPS | **0.22 (0.004)** | 0.09 (0.005) | -0.07 (0.15) | -0.07 (0.03) | **0.18 (0.06)** | 0.06 (0.16) |
| Anti-inflammatory index | LD-LPS | **0.25 (0.004)** | 0.14 (0.006) | 0.16 (0.17) | -0.07 (0.03) | **0.22 (0.07)** | 0.05 (0.18) |
|  | HD-LPS | **0.20 (0.004)** | 0.14 (0.006) | **0.20 (0.15)** | -0.08 (0.03) | 0.12 (0.06) | 0.14 (0.16) |
| Ratio pro/anti inflammatory index | LD-LPS | 0.002 (0.04)  ♂ 0.05 (0.05)  ♀-0.09 (0.05) | -0.05 (0.05)  ♂ -0.29 (0.06)  ♀ 0.04 (0.07) | -0.15 (1.40) | 0.006 (0.28) | 0.009 (0.55)  ♂ 0.08 (0.71)  ♀-0.21 (0.73) | -0.03 (1.48) |
|  | HD-LPS | **-0.21 (0.04)** | -0.11 (0.06) | -0.02 (1.51) | -0.03 (0.30) | 0.06 (0.59) | -0.02 (1.58) |
| TNF-α | LD-LPS | 0.15 (0.005) | 0.07 (0.007) | -0.08 (0.18) | -0.03 (0.04)  **♂ 0.34 (0.26)**  ♀ -0.07 (0.03) | **0.20 (0.07)** | -0.08 (0.19) |
|  | HD-LPS | 0.19 (0.004) | 0.10 (0.005) | -0.14 (0.15) | -0.04 (0.030)  ♂ 0.38 (0.15)  ♀ -0.07 (0.03) | 0.10 (0.06) | 0.009 (0.16) |
| IL-1β | LD-LPS | **0.20 (0.005)** | 0.14 (0.008) | 0.09 (0.21) | -0.03 (0.04) | 0.18 (0.08) | -0.008 (0.22) |
|  | HD-LPS | **0.22 (0.004)** | 0.13 (0.005) | 0.04 (0.15) | -0.02 (0.03) | 0.15 (0.06) | 0.10 (0.16) |
| IL-1RA | LD-LPS | **0.23 (0.003)** | 0.09 (0.005)  ♂ -0.03 (0.01)  **♀ 0.16 (0.004)** | **0.18 (0.12)** | 0.04 (0.02) | **0.33 (0.05)** | 0.05 (0.30) |
|  | HD-LPS | **0.21 (0.002)** | 0.12 (0.004) | **0.23 (0.10)** | -0.01 (0.02) | **0.27 (0.04)** | 0.09 (0.10) |
| IL-4 | LD-LPS | - | **-** | - | - | - | - |
|  | HD-LPS | - | **-** | - | - | - | **-** |
| IL-10 | LD-LPS | **0.19 (0.004)** | 0.15 (0.006) | 0.08 (0.17) | -0.16 (0.03) | 0.05 (0.06) | 0.04 (0.17) |
|  | HD-LPS | 0.11 (0.004) | 0.10 (0.005) | 0.10 (0.14) | -0.12 (0.03) | -0.08 (0.05) | 0.13 (0.15) |
| CCL2 | LD-LPS | 0.04 (0.004) | -0.10 (0.005) | -0.06 (0.14) | -0.04 (0.03)  **♂ 0.45 (0.24)**  ♀ -0.08 (0.03) | 0.02 (0.05) | 0.05 (0.15) |
|  | HD-LPS | 0.06 (0.004) | 0.006 (0.005) | -0.05 (0.14) | -0.05 (0.03) | 0.04 (0.05) | -0.05 (0.14) |
| CCL3 | LD-LPS | 0.08 (0.005) | -0.03 (0.007) | 0.02 (0.20) | -0.05 (0.04)  ♂ 0.41 (0.21)  ♀-0.19 (0.20) | **0.21 (0.07)** | -0.004 (0.20) |
|  | HD-LPS | 0.15 (0.003) | 0.06 (0.005) | -0.07 (0.13) | -0.07 (0.03) | 0.15 (0.05) | 0.06 (0.14) |
| CCL4 | LD-LPS | 0.12 (0.004) | -0.04 (0.006) | -0.004 (0.16) | -0.05 (0.03) | **0.25 (0.06)** | 0.002 (0.16) |
|  | HD-LPS | **0.21 (0.003)** | 0.04 (0.004) | -0.03 (0.10) | -0.08 (0.02) | **0.25 (0.04)** | 0.09 (0.11) |
| **Ex-vivo** | | | | | | |  |
| Inflammatory index |  | **0.23 (0.003)** | -0.01 (0.005) | -0.007 (0.14) | -0.12 (0.13) | 0.09 (0.05) | -0.06 (0.15) |
| Pro inflammatory index |  | 0.17 (0.004) | -0.11 (0.005) | -0.08 (0.15) | -0.14 (0.14) | 0.05 (0.06) | 0.08 (0.16) |
| Anti-inflammatory index |  | **0.22 (0.004)** | 0.07 (0.006) | 0.06 (0.17) | -0.07 (0.16) | 0.10 (0.07) | -0.16 (0.18) |
| Ratio pro/ anti inflammatory index |  | 0.06 (0.15) | 0.006 (0.21) | 0.09 (5.73) | -0.14 (0.14) | -0.15 (2.28) | -0.14 (6.03) |
| hsCRP |  | 0.16 (0.006) | -0.14 (0.009) | 0.04 (0.25) | -0.16 (0.24) | 0.07 (0.098) | **0.18 (0.25)** |
| TNF-α |  | 0.07 (0.003)  ♂ 0.14 (0.006)  ♀ 0.06 (0.003) | -0.03 (0.004) | **-0.19 (0.12)** | -0.09 (0.12) | -0.03 (0.05) | -0.03 (0.13) |
| sTNF-R2 |  | **0.23 (0.002)** | 0.07 (0.003) | -0.02 (0.08) | -0.03 (0.08) | 0.13 (0.03) | -0.11 (0.09) |
| Il-1β |  | 0.16 (0.01) | 0.09 (0.02) | -0.15 (0.49) | 0.03 (0.47) | -0.33 (0.18) | -0.08 (0.50) |
| IL-1RA |  | 0.13 (0.003) | 0.06 (0.004) | 0.12 (0.11) | -0.09 (0.10) | 0.03 (0.04) | -0.16 (0.11) |

Data represents standardized regression coefficient β (standard error, unstandardized B) of various lifestyle factors demonstrating an association with *ex-vivo* and *in-vitro* inflammatory indexes and of *in-vitro* and *ex-vivo* single inflammatory mediators using linear regression analysis. All single neuroimmune responses are ln-transformed. The *in-vitro* neuroimmune responses are normalized for monocyte count. Significant values are in bold font (^*^p < 0.05) TNF-α, tumor necrosis factor -α; IL-1β, interleukin -1β; IL-1RA, interleukin -receptor antagonist; IL-4, interleukin – 4; IL-10, interleukin -10; CCL2 / MCP1, c-c-motif ligand 2 also referred to as monocyte chemoattractant protein 1; CCL3 / MIP1α, c-c-motif ligand 3 also referred to as macrophage inflammatory protein 1α; CCL4 / MIP1β, c-c-motif ligand 4 also referred to as macrophage inflammatory protein 1β; TNFα : IL-10, ratio between TNFα : IL-10; IL-1β : IL-1RA, ratio between IL-1β : IL-1RA; 1ng/ml (LD-LPS), 1 milliliter whole-blood stimulation with 1 nanogram TLR4 agonist lipopolysacharide; 10µg/ml (HD-LPS), 1 milliliter whole blood stimulation with 10 microgram of TLR4 agonist lipopolysacharide; ♂ effect modification for males; ♀ effect modification for females.

^a^Refers to pain intensity measured on the visual analogue scale, 0-100

^b^Refers to score on the neck disability index, 0-100

^c^Male:0 Female:1

^d^Refers to total score on the central sensitisation questionnaire higher than 40

^e^Refers having insomnia (pittsburg sleep quality index score above 5)

**SUPPLEMENTARY F3** Association of psychological factors with neuroimmune responses in people with non-specific neck pain.

|  | | **Mental Health^a^** | **Stress^b^** | **Depression^c^** | **Anxiety^d^** | **Kinesiophobia^e^** | **Pain rumination** | **Pain magnification** | **Pain helplessness** | **Catastrophizing^jf^** |
| --- | --- | --- | --- | --- | --- | --- | --- | --- | --- | --- |
|  | | **Standardized- adjusted** β **(SE)** | **Standardized- adjusted** β **(SE)** | **Standardized- adjusted** β **(SE)** | **Standardized- adjusted** β **(SE)** | **Standardized- adjusted** β **(SE)** | **Standardized- adjusted** β **(SE)** | **Standardized- adjusted** β **(SE)** | **Standardized- adjusted** β **(SE)** | **Standardized- adjusted** β **(SE)** |
| **In-vitro** | | | | | | | | | | |
| Inflammatory index | LD-LPS | -0.06 (0.005) | 0.15 (0.009) | 0.04 (0.009) | **0.23 (0.01)** | 0.03 (0.17) | 0.06 (0.02) | 0.03 (0.03) | -0.03 (0.01) | 0.03 (0.007) |
|  | HD-LPS | -0.03 (0.004) | 0.003 (0.008) | -0.08 (0.008) | 0.13 (0.009) | 0.05 (0.15) | 0.11 (0.02) | 0.11 (0.02) | 0.06 (0.01) | 0.10 (0.006) |
| Pro-inflammatory index | LD-LPS | -0.04 (0.005) | 0.14 (0.009) | 0.01 (0.009) | **0.19 (0.01)** | 0.007 (0.17) | 0.04 (0.02) | -0.006 (0.03) | -0.07 (0.01) | -0.005 (0.007) |
|  | HD-LPS | -0.01 (0.004) | 0.004 (0.008) | -0.11 (0.008) | 0.10 (0.01) | 0.06 (0.16) | 0.13 (0.02) | 0.07 (0.02) | 0.04 (0.01) | 0.08 (0.006) |
| Anti-inflammatory index | LD-LPS | -0.10 (0.005) | 0.15 (0.009) | 0.11 (0.009) | **0.18 (0.01)** | 0.07 (0.18) | 0.09 (0.02) | 0.11 (0.03) | 0.05 (0.02) | 0.10 (0.007) |
|  | HD-LPS | -0.07 (0.005) | -0.002 (0.009) | 0.008 (0.009) | 0.17 (0.01) | 0.02 (0.17) | 0.06 (0.02) | 0.16 (0.03) | 0.08 (0.01) | 0.10 (0.007) |
| Ratio pro/anti inflammatory index | LD-LPS | 0.14 (0.04) | -0.17 (0.08) | -0.05 (0.08) | -0.14 (0.09) | 0.04 (1.49) | 0.005 (0.17)  ♂ 0.05 (0.22)  ♀-0.25 (0.22) | 0.001 (0.24) | 0.01 (0.13)  ♂ 0.07 (0.17)  ♀ -0.09 (0.18) | 0.002 (0.06) |
|  | HD-LPS | 0.06 (0.04) | 0.05 (0.08) | **-0.24 (0.08)** | -0.16 (0.10) | 0.03 (1.58) | -0.15 (0.18) | -0.11 (0.25) | -0.17 (0.13) | -0.17 (0.06) |
| TNF-α | LD-LPS | 0.06 (0.005) | 0.08 (0.01) | -0.04 (0.01)  ♂ 0.22 (0.03)  **♀ -0.12 (0.01)** | 0.12 (0.01) | 0.06 (0.19) | 0.07 (0.02) | -0.02 (0.03)  ♂ 0.27 (0.07)  **♀ -0.13 (0.04)** | -0.04 (0.02) | 0.02 (0.008) |
|  | HD-LPS | 0.02 (0.004) | 0.04 (0.008) | -0.12 (0.008) | 0.08 (0.01) | 0.06 (0.16) | 0.15 (0.02 ) | 0.06 (0.03) | 0.07 (0.01)  **♂ 0.18 (0.03)**  ♀ 0.06 (0.02) | 0.10 (0.006) |
| IL-1β | LD-LPS | -0.20 (0.006) | 0.17 (0.01) | 0.08 (0.01) | 0.18 (0.01) | 0.14 (0.22) | 0.15 (0.03) | 0.007 (0.04) | 0.03 (0.02) | 0.08 (0.009) |
|  | HD-LPS | -0.04 (0.004) | -0.03 (0.008) | -0.11 (0.008) | 0.04 (0.01) | 0.08 (0.16) | 0.17 (0.02) | 0.06 (0.02) | 0.09 (0.01) | 0.12 (0.006) |
| IL-1RA | LD-LPS | -0.10 (0.004)  ♂ -0.25 (0.01)  ♀ 0.03 (0.003) | 0.05 (0.007) | 0.02 (0.007) | 0.18 (0.008) | 0.005 (0.13) | 0.08 (0.02) | 0.07 (0.02) | 0.07 (0.01) | 0.10 (0.005) |
|  | HD-LPS | -0.05 (0.003)  ♂ -0.17 (0.006)  ♀0.06 (0.003) | -0.16 (0.005) | -0.08 (0.005) | 0.08 (0.007) | -0.01 (0.10) | 0.12 (0.01) | 0.12 (0.02) | 0.12 (0.009) | 0.14 (0.004) |
| IL-4 | LD-LPS | - | - | - | - | - | - | - | - | - |
|  | HD-LPS | - | - | - | - | - | - | - | - | - |
| IL-10 | LD-LPS | -0.07 (0.005) | **0.21 (0.009)** | 0.17 (0.009) | **0.29 (0.01)** | 0.11 (0.17) | 0.06 (0.02) | 0.12 (0.03) | 0.01 (0.02) | 0.07 (0.007) |
|  | HD-LPS | -0.07 (0.004) | 0.16 (0.008) | 0.09 (0.008) | **0.19 (0.009)** | 0.04 (0.15) | -0.02 (0.02) | 0.14 (0.02) | 0.008 (0.01) | 0.03 (0.006) |
| CCL2 | LD-LPS | 0.01 (0.004) | 0.05 (0.008) | 0.008 (0.008)  ♂ 0.52 (0.02)  ♀ -0.04 (0.008) | 0.13 (0.009) | -0.07 (0.15) | -0.04 (0.02) | 0.04 (0.03) | -0.03 (0.01) | -0.03 (0.006) |
|  | HD-LPS | -0.04 (0.004) | 0.06 (0.008) | 0.09 (0.007)  ♂ 0.32 (0.02)  ♀ -0.08 (0.008) | 0.08 (0.009) | 0.08 (0.14) | 0.01 (0.02) | 0.03 (0.02) | 0.03 (0.01) | 0.03 (0.006) |
| CCL3 | LD-LPS | -0.003 (0.006) | 0.12 (0.01) | 0.001 (0.01)  ♂ 0.29 (0.04)  **♀ -0.16 (0.009)** | 0.15 (0.01) | -0.03 (0.21) | 0.003 (0.02) | -0.05 (0.03) | -0.11 (0.02) | -0.05 (0.009) |
|  | HD-LPS | 0.04 (0.004) | -0.01 (0.007) | -0.16 (0.007) | 0.06 (0.009) | 0.02 (0.14) | 0.08 (0.02) | 0.04 (0.02) | -0.02 (0.01) | 0.03 (0.005) |
| CCL4 | LD-LPS | -0.02 (0.005) | 0.11 (0.009) | 0.005 (0.009)  ♂ 0.26 (0.03)  **♀-0.13 (0.007)** | **0.18 (0.010)** | -0.07 (0.17) | -0.01 (0.02) | -0.002 (0.03) | -0.10 (0.01) | -0.05 (0.007) |
|  | HD-LPS | -0.01 (0.003) | -0.04 (0.006) | -0.12 (0.005) | 0.13 (0.007) | -0.001 (0.11) | 0.06 (0.01) | 0.11 (0.02) | -0.01 (0.009) | 0.05 (0.004) |
| **Ex-vivo** | | | | | | | | | | |
| Inflammatory index |  | 0.18 (0.004) | -0.19 (0.007) | -0.14 (0.007) | -0.05 (0.009) | -0.09 (0.14) | **-0.21 (0.02)** | -0.15 (0.02) | -0.17 (0.01) | **-0.20 (0.006)** |
| Pro inflammatory index |  | 0.18 (0.004) | -0.19 (0.008) | -0.15 (0.008) | -0.08 (0.01) | -0.08 (0.16) | **-0.27 (0.02)** | **-0.25 (0.02)** | **-0.26 (0.01)** | **-0.28 (0.006)** |
| Anti-inflammatory index |  | 0.13 (0.005) | -0.14 (0.009) | -0.09 (0.009) | -0.02 (0.01) | -0.08 (0.17) | -0.11 (0.02) | -0.02 (0.03) | -0.05 (0.02) | -0.08 (0.007) |
| Ratio pro/ anti inflammatory index |  | 0.09 (0.16) | 0.06 (0.31) | 0.04 (0.30) | -0.008 (0.39) | 0.09 (5.99) | -0.02 (0.69) | 0.05 (0.95) | 0.05 (0.51) | 0.03 (0.25) |
| hsCRP |  | **0.20 (0.007)** | **-0.19 (0.01)** | -0.09 (0.01) | -0.10 (0.02) | 0.01 (0.26) | **-0.25 (0.03)** | **-0.20 (0.04)** | -0.18 (0.02) | **-0.23 (0.01)** |
| TNF-α |  | 0.09 (0.003)  ♂**0.28 (0.009)**  ♀-0.05 ().004) | -0.11 (0.007) | -0.15 (0.006) | -0.04 (0.008) | -0.14 (0.126) | -0.17 (0.01)  **♂-0.15 (0.03)**  ♀-0.20 (0.02) | -0.17 (0.02) | **-0.23 (0.01)** | **-0.21 (0.005)** |
| sTNF-R2 |  | 0.07 (0.002) | -0.11 (0.004) | -0.07 (0.004) | -0.02 (0.005) | -0.09 (0.08) | -0.05 (0.01) | -0.04 (0.01) | -0.09 (0.007) | -0.06 (0.003) |
| Il-1β |  | 0.27 (0.02) | -0.12 (0.02) | -0.09 (0.02) | -0.25 (0.03) | -0.23 (0.59) | 0.002 (0.49) | -0.16 (0.07) | -0.24 (0.09) | -0.12 (0.05) |
| IL-1RA |  | 0.15 (0.003) | -0.11 (0.006) | -0.08 (0.006) | -0.01 (0.007) | -0.04 (0.11) | -0.12 (0.01) | 0.006 (0.02) | 0.01 (0.01) | -0.06 (0.005) |

Data represent standardized regression coefficient-β (standard error, unstandardized B) of various psychological factors demonstrating an association with several neuroimmune responses *ex-vivo* and after *in-vitro* whole blood stimulation using linear regression models. All neuroimmune responses are Ln-transformed and *in-vitro* responses are normalized for monocyte count. Significant values are in bold font (^*^p < 0.05). TNF-α, tumor necrosis factor -α; IL-1β, interleukin -1β; IL-1RA, interleukin -receptor antagonist; IL-4, interleukin – 4; IL-10, interleukin -10; CCL2 / MCP1, c-c-motif ligand 2 also referred to as monocyte chemoattractant protein 1; CCL3 / MIP1α, c-c-motif ligand 3 also referred to as macrophage inflammatory protein 1α; CCL4 / MIP1β, c-c-motif ligand 4 also referred to as macrophage inflammatory protein 1β; TNFα : IL-10, ratio between TNFα : IL-10; IL-1β : IL-1RA, ratio between IL-1β : IL-1RA; 1ng/ml (LD-LPS), 1 milliliter whole-blood stimulation with 1 nanogram TLR4 agonist lipopolysacharide; 10µg/ml (HD-LPS), 1 milliliter whole blood stimulation with 10 microgram of TLR4 agonist lipopolysacharide; ♂ effect modification for males; ♀ effect modification for females.

^a^Refers to mental health inventory -5 score

^b^Refers to score on the depression, anxiety and stress scale 21 subsection stress

^c^Refers to the score on the depression, anxiety and stress scale 21 subsection depression

^d^Refers to the score on the depression, anxiety and stress scale 21 subsection anxiety

^e^Refers to total score on the tampa scale questionnaire higher than 37

^f^Refers to the total score on the pain catastrophizing scale

**SUPPLEMENTARY G1** Association of lifestyle factors with neuroimmune responses in people with a cervical radiculopathy

|  | | **Physical Activity^a^** | **Smoking^b^** | **Alcohol use^c^** | **BMI** | **Visceral Adipose^d^** |
| --- | --- | --- | --- | --- | --- | --- |
|  | | **Standardized- adjusted** β **(SE)** | **Standardized- adjusted** β **(SE)** | **Standardized- adjusted** β **(SE)** | **Standardized- adjusted** β **(SE)** | **Standardized- adjusted** β **(SE)** |
| **In-vitro** | | | | | | |
| Inflammatory index | LD-LPS | -0.18 (0.02) | 0.19 (0.34) | 0.03 (0.35) | 0.12 (0.04) | 0.10 (0.005) |
|  | HD-LPS | -0.13 (0.02) | 0.07 (0.32) | -0.17 (0.32) | 0.22 (0.03)  ♂ 0.25 (0.06)  ♀ 0.15 (0.06) | 0.14 (0.005)  ♂ -0.09 (0.01)  ♀ 0.16 (0.01) |
| Pro-inflammatory index | LD-LPS | -0.23 (0.02) | 0.15 (0.35) | 0.13 (0.35) | 0.11 (0.04) | 0.09 (0.006) |
|  | HD-LPS | -0.17 (0.02) | -0.06 (0.34) | -0.04 (0.34) | 0.29 (0.03) | 0.20 (0.005) |
| Anti-inflammatory index | LD-LPS | -0.05 (0.03) | 0.25 (0.38) | -0.19 (0.39) | 0.13 (0.04) | 0.11 (0.006) |
|  | HD-LPS | -0.01 (0.02) | 0.35 (0.34) | -0.42 (0.34) | 0.008 (0.04)  ♂ 0.26 (0.07)  ♀ -0.35 (0.06) | -0.04 (0.006)  ♂ -0.002 (0.01)  ♀ -0.33 (0.01) |
| Ratio pro/anti inflammatory index | LD-LPS | -0.25 (0.11) | -0.16 (1.62) | 0.04 (1.6) | -0.20 (0.16) | -0.13 (0.03) |
|  | HD-LPS | 0.07 (0.22) | -0.24 (3.28) | 0.22 (3.33) | 0.12 (0.34) | 0.19 (0.05) |
| TNF-α | LD-LPS | -0.15 (0.04) | -0.11 (0.53) | 0.14 (0.53) | 0.29 (0.05) | 0.27 (0.008) |
|  | HD-LPS | 0.02 (0.03) | **-0.51 (0.37)** | 0.06 (0.43) | 0.40 (0.04) | 0.32 (0.006) |
| IL-1β | LD-LPS | -0.23 (0.04) | 0.04 (0.54) | -0.05 (0.55) | 0.14 (0.05) | 0.05 (0.009) |
|  | HD-LPS | -0.16 (0.02) | -0.17 (0.31) | -0.13 (0.31) | 0.29 (0.03) | 0.17 (0.005) |
| IL-1RA | LD-LPS | -0.11 (0.02) | 0.07 (0.25) | -0.15 (0.25) | 0.37 (0.02) | 0.31 (0.004) |
|  | HD-LPS | -0.05 (0.01) | 0.22 (0.20) | -0.38 (0.19) | 0.35 (0.02) | 0.26 (0.003) |
| IL-4 | LD-LPS | - | - | - | - | - |
|  | HD-LPS | **-** | **-** | **-** | **-** | **-** |
| IL-10 | LD-LPS | 0.02 (0.03) | 0.36 (0.37) | -0.18 (0.39) | -0.15 (0.04) | -0.13 (0.006) |
|  | HD-LPS | 0.03 (0.02) | 0.32 (0.035) | -0.27 (0.36) | -0.33 (0.03)  ♂ -0.53 (0.07)  **♀ -0.39 (0.05)** | -0.31 (0.005)  ♂ -0.51 (0.01)  **♀ -0.47 (0.008)** |
| CCL2 | LD-LPS | -0.14 (0.02) | **0.50 (0.26)** | 0.06 (0.30) | -0.29 (0.03)  ♂ -0.71 (0.02)  ♀ -0.12 (0.05) | -0.19 (0.005) |
|  | HD-LPS | -0.32 (0.02) | **0.63 (0.29)** | -0.02 (0.39) | -0.19 (0.04) | -0.12 (0.006) |
| CCL3 | LD-LPS | -0.29 (0.02) | -0.02 (0.36) | 0.19 (0.36) | 0.09 (0.04) | 0.04 (0.006) |
|  | HD-LPS | -0.08 (-0.006) | -0.30 (0.24) | 0.02 (0.26) | 0.28 (0.03) | 0.16 (0.004) |
| CCL4 | LD-LPS | -0.05 (0.02) | 0.14 (0.28) | 0.14 (0.29) | 0.19 (0.03) | 0.15 (0.004) |
|  | HD-LPS | -0.06 (0.01)  ♂ -0.003 (0.02)  ♀ -0.07 (0.05) | 0.14 (0.21) | -0.07 (0.21) | 0.26 (0.02) | 0.17 (0.003) |
| **Ex-vivo** | | | | | | |
| Inflammatory index |  | -0.23 (0.02) | -0.10 (0.27) | -0.33 (0.26) | 0.31 (0.03) | **0.43 (0.004)** |
| Pro-inflammatory index |  | -0.07 (0.02) | -0.16 (0.21) | -0.33 (0.21) | 0.38 (0.02) | 0.37 (0.003) |
| Anti-inflammatory index |  | -0.27 (0.03) | -0.04 (0.38) | -0.28 (0.38) | 0.22 (0.04) | 0.39 (0.005) |
| Ratio pro/anti inflammatory index |  | -0.13 (0.33) | 0.29 (4.72) | 0.29 (4.72) | -0.34 (0.47) | -0.33 (0.07) |
| hsCRP |  | -0.11 (0.03) | -0.10 (0.39) | -0.26 (0.39) | **0. 36 (0.04)** | 0.29 (0.01) |
| TNF-α |  | **-0.41 (0.01)** | -0.19 (0.20) | -0.03 (0.22) | **0.21 (0.02)** | 0.40 (0.003) |
| sTNF-R2 |  | -0.28 (0.01) | 0.02 (0.11) | -0.18 (0.11) | 0.18 (0.01) | 0.38 (0.002) |
| Il-1β |  | - | - | - | - | - |
| IL-1RA |  | -0.20 (0.01) | -0.10 (0.19) | -0.32 (0.18) | 0.20 (0.02) | -0.17 (0.03) |

Data represent standardized regression coefficient β (standard error, unstandardized B) of various lifestyle factors demonstrating an association with *ex-vivo* and *in-vitro* inflammatory indexes and of *in-vitro* and *ex-vivo* single inflammatory mediators using linear regression analysis. All single neuroimmune responses are ln-transformed. The *in-vitro* neuroimmune responses are normalized for monocyte count. Significant values are in bold font (^*^p < 0.05). TNF-α, tumor necrosis factor -α; IL-1β, interleukin -1β; IL-1RA, interleukin -receptor antagonist; IL-4, interleukin – 4; IL-10, interleukin -10; CCL2 / MCP1, c-c-motif ligand 2 also referred to as monocyte chemoattractant protein 1; CCL3 / MIP1α, c-c-motif ligand 3 also referred to as macrophage inflammatory protein 1α; CCL4 / MIP1β, c-c-motif ligand 4 also referred to as macrophage inflammatory protein 1β; TNFα : IL-10, ratio between TNFα : IL-10; IL-1β : IL-1RA, ratio between IL-1β : IL-1RA; 1ng/ml (LD-LPS), 1 milliliter whole-blood stimulation with 1 nanogram TLR4 agonist lipopolysacharide; 10µg/ml (HD-LPS), 1 milliliter whole blood stimulation with 10 microgram of TLR4 agonist lipopolysacharide; ♂ effect modification for males; ♀ effect modification for females.

^a^Refers to 1000/metabolic equivalent of a task

^b^Refers to current smoker, yes:1, no:0

^c^Refers to consuming alcohol, yes:1, no:0

^d^Refers to linear distance in millimeters between the posterior aspect of peritoneum and anterior aspect of lumbar vertebra T10

**SUPPLEMENTARY G2** Association of clinical factors with neuroimmune responses in people with a cervical radiculopathy

|  | | **Pain intensity^a^** | **Disability^b^** | **Sex^c^** | | **Central Sensitization^d^** | **Number of co-morbidities** | | **Insomnia^e^** | |
| --- | --- | --- | --- | --- | --- | --- | --- | --- | --- | --- |
|  | | **Standardized- adjusted** β **(SE)** | **Standardized- adjusted** β **(SE)** | **Standardized- adjusted** β **(SE)** | **Standardized- adjusted** β **(SE)** | | | **Standardized- adjusted** β **(SE)** | **Standardized- adjusted** β **(SE)** | |
| **In-vitro** | | | | | | | | |  |  |
| Inflammatory index | LD-LPS | 0.25 (0.01) | 0.2 (0.01) | -0.10 (0.35) | -0.19 (0.37) | | | 0.04 (0.14) | -0.30 (0.37) |  |
|  | HD-LPS | 0.15 (0.01)  ♂ 0.30 (0.01)  ♀ 0.05 (0.03) | **0.42 (0.01)**  ♂ 0.20 (0.01)  ♀ 0.77 (0.02) | -0.31 (0.31) | 0.11 (0.34) | | | 0.16 (0.12) | -0.27 (0.33) |  |
| Pro-inflammatory index | LD-LPS | 0.23 (0.01) | 0.01 (0.01) | -0.10 (0.36) | -0.15 (0.38) | | | 0.10 (0.14) | -0.25 (0.37) |  |
|  | HD-LPS | 0.15 (0.01) | 0.36 (0.01)  ♂ 0.13 (0.01)  ♀ 0.74 (0.02) | -0.29 (0.33) | 0.11 (0.36) | | | 0.17 (0.13) | -0.18 (0.36) |  |
| Anti-inflammatory index | LD-LPS | 0.16 (0.01) | 0.04 (0.01) | -0.10 (0.40) | -0.27 (0.41) | | | -0.11 (0.16) | -0.36 (0.40) |  |
|  | HD-LPS | 0.11 (0.01)  ♂ 0.35 (0.01)  ♀ -0.14 (0.03) | **0.47 (0.01)**  ♂ 0.28 (0.01)  ♀ 0.67 (0.02) | -0.19 (0.36) | 0.10 (0.39)  ♂ -0.46 (0.38)  ♀ **1.16 (0.57)** | | | 0.10 (0.14) | -0.41 (0.36) |  |
| Ratio pro/anti inflammatory index | LD-LPS | -0.13 (0.06) | -0.06 (0.06) | 0.19 (1.64) | -0.03 (1.77) | | | -0.17 (0.39) | -0.23 (1.74) |  |
|  | HD-LPS | -0.29 (0.12) | **-0.47 (0.10)** | -0.17 (3.39) | -0.24 (3.54) | | | 0.15 (1.34) | 0.06 (3.68) |  |
| TNF-α | LD-LPS | 0.37 (0.02) | -0.07 (0.02) | 0.08 (0.54) | -0.46 (0.52) | | | -0.26 (0.20) | -0.06 (0.58) |  |
|  | HD-LPS | 0.03 (0.02) | 0.18 (0.01) | -0.05 (0.43) | -0.16 (0.45) | | | 0.05 (0.17) | -0.007 (0.46) |  |
| IL-1β | LD-LPS | 0.38 (0.02) | -0.06 (0.02) | 0.11 (0.55) | -0.38 (0.55) | | | 0.01 (0.22) | -0.09 (0.59) |  |
|  | HD-LPS | 0.13 (0.01) | 0.29 (0.01) | -0.12 (0.31) | 0.02 (0.33) | | | 0.16 (0.12) | -0.02 (0.34) |  |
| IL-1RA | LD-LPS | 0.32 (0.009) | 0.22 (0.008) | -0.21 (0.25) | -0.42 (0.25)  ♂ 0.98 (0.24)  ♀ -0.62 (0.26) | | | -0.25 (0.09) | -0.32 (0.26) |  |
|  | HD-LPS | **0.51 (0.006)** | **0.54 (0.006)** | -0.23 (0.20) | -0.29 (0.21) | | | -0.09 (0.08) | -0.34 (0.21) |  |
| IL-4 | LD-LPS | - | **-** | - | - | | | - | - |  |
|  | HD-LPS | - | **-** | - | - | | | - | **-** |  |
| IL-10 | LD-LPS | 0.12 (0.02) | -0.15 (0.01) | 0.04 (0.41) | -0.05 (0.43) | | | 0.06 (0.16) | -0.30 (0.42) |  |
|  | HD-LPS | -0.34 (0.01) | 0.19 (0.01) | -0.22 (0.37) | 0.45 (0.36)  ♂ 1.07 (0.85)  ♀ 0.04 (0.35) | | | 0.24 (0.14) | -0.30 (0.38) |  |
| CCL2 | LD-LPS | -0.22 (0.01) | 0.31 (0.01) | -0.28 (0.29) | **0.59 (0.27)**  ♂ 0.57 (0.62)  ♀ | | | **0.49 (0.10)** | -0.30 (0.31) |  |
|  | HD-LPS | 0.23 (0.01) | 0.10 (0.01) | -0.18 (0.39) | 0.13 (0.41) | | | 0.08 (0.15) | -0.19 (0.41) |  |
| CCL3 | LD-LPS | 0.15 (0.01) | -0.16 (0.01) | -0.02 (0.37) | -0.24 (0.38) | | | 0.12 (0.14) | -0.18 (0.39) |  |
|  | HD-LPS | -0.07 (0.009) | 0.27 (0.008) | -0.29 (0.25) | 0.13 (0.27) | | | 0.27 (0.09) | -0.09 (0.28) |  |
| CCL4 | LD-LPS | 0.18 (0.01) | 0.03 (0.01) | -0.25 (0.28) | -0.06 (0.31) | | | 0.01 (0.12) | -0.32 (0.30) |  |
|  | HD-LPS | 0.21 (0.007) | **0.44 (0.006)**  ♂ 0.93 (0.009)  ♀ 0.29 (0.008) | -0.39 (0.20) | 0.26 (0.22)  ♂ 1.11 (0.36)  ♀ -0.04 (0.27) | | | 0.04 (0.08) | -0.32 (0.22) |  |
| **Ex-vivo** | | | | | | | | |  |  |
| Inflammatory index |  | 0.01 (0.22) | 0.11 (0.01) | -0.04 (0.28) | -0.34 (0.26) | | | -0.28 (0.11) | -0.03 (0.29) |  |
| Pro-inflammatory index |  | **0.42 (0.008)** | 0.32 (0.008) | -0.007 (0.22) | -0.29 (0.21) | | | -0.25 (0.09) | 0.16 (0.23) |  |
| Anti-inflammatory index |  | 0.08 (0.02) | -0.01 (0.01) | -0.06 (0.39) | -0.30 (0.37) | | | -0.25 (0.16) | -0.13 (0.41) |  |
| Ratio pro/anti inflammatory index |  | -0.17 (0.18) | -0.25 (0.17) | 0.43 (4.38) | -0.29 (0.21) | | | 0.26 (1.89) | 0.03 (5.21) |  |
| hsCRP |  | **0.45 (0.01)** | 0.38 (0.01) | 0.05 (0.41) | -0.29 (0.38) | | | -0.21 (0.16) | **0.13 (0.43)** |  |
| TNF-α |  | 0.05 (0.01) | 0.07 (0.01) | -0.10 (0.21) | -0.24 (0.21) | | | -0.32 (0.08) | 0.02 (0.23) |  |
| sTNF-R2 |  | -0.16 (0.004) | -0.13 (0.004) | -0.10 (0.11) | -0.28 (0.11) | | | -0.21 (0.05) | -0.24 (0.11) |  |
| Il-1β |  | - | - | - | - | | | - | - |  |
| IL-1RA |  | -0.30 (0.01) | 0.10 (0.01) | 0.01 (0.19) | -0.26 (0.19) | | | -0.23 (0.08) | 0.01 (0.20) |  |

Data represent standardized regression coefficient β (standard error, unstandardized B) of various clinical factors demonstrating an association with *ex-vivo* and *in-vitro* inflammatory indexes and of *in-vitro* and *ex-vivo* single inflammatory mediators using linear regression analysis. All single neuroimmune responses are ln-transformed. The *in-vitro* neuroimmune responses are normalized for monocyte count. Significant values are in bold font (^*^p < 0.05) TNF-α, tumor necrosis factor -α; IL-1β, interleukin -1β; IL-1RA, interleukin -receptor antagonist; IL-4, interleukin – 4; IL-10, interleukin -10; CCL2 / MCP1, c-c-motif ligand 2 also referred to as monocyte chemoattractant protein 1; CCL3 / MIP1α, c-c-motif ligand 3 also referred to as macrophage inflammatory protein 1α; CCL4 / MIP1β, c-c-motif ligand 4 also referred to as macrophage inflammatory protein 1β; TNFα : IL-10, ratio between TNFα : IL-10; IL-1β : IL-1RA, ratio between IL-1β : IL-1RA; 1ng/ml (LD-LPS), 1 milliliter whole-blood stimulation with 1 nanogram TLR4 agonist lipopolysacharide; 10µg/ml (HD-LPS), 1 milliliter whole blood stimulation with 10 microgram of TLR4 agonist lipopolysacharide; ♂ effect modification for males; ♀ effect modification for females.

^a^Refers to pain intensity measured on the visual analogue scale, 0-100

^b^Refers to score on the neck disability index, 0-100

^c^Male:0 Female:1

^d^Refers to total score on the central sensitisation questionnaire higher than 40

^e^Refers having insomnia (pittsburg sleep quality index score above 5)

**SUPPLEMENTARY G3** Association of psychological factors with neuroimmune responses in people with a cervical radiculopathy

|  | | **Mental Health^a^** | **Stress^b^** | **Depression^c^** | **Anxiety^d^** | **Kinesiophobia^e^** | **Pain rumination** | **Pain magnification** | **Pain helplessness** | **Catastrophizing^f^** |
| --- | --- | --- | --- | --- | --- | --- | --- | --- | --- | --- |
|  | | **Standardized- adjusted** β **(SE)** | **Standardized- adjusted** β **(SE)** | **Standardized- adjusted** β **(SE)** | **Standardized- adjusted** β **(SE)** | **Standardized- adjusted** β **(SE)** | **Standardized- adjusted** β **(SE)** | **Standardized- adjusted** β **(SE)** | **Standardized- adjusted** β **(SE)** | **Standardized- adjusted** β **(SE)** |
| **In-vitro** | | | | | | | | | | |
| Inflammatory index | LD-LPS | -0.13 (0.01) | -0.02 (0.02) | 0.10 (0.02) | 0.07 (0.02)  ♂ -0.41 (0.03)  ♀ **0.93 (0.02)** | 0.32 (0.37)  ♂ 0.27 (0.52)  ♀ **0.82 (0.56)** | 0.27 (0.04) | **0.53 (0.06)** | 0.05 (0.03) | 0.28 (0.02) |
|  | HD-LPS | -0.11 (0.01) | 0.23 (0.02) | 0.25 (0.02) | 0.24 (0.02)  ♂ -0.25 (0.02)  ♀ **0.77 (0.02)** | 0.37 (0.33)  ♂ 0.26 (0.39)  ♀ **0.75 (0.55)** | 0.14 (0.04) | 0.15 (0.06) | 0.33 (0.03) | 0.28 (0.01) |
| Pro-inflammatory index | LD-LPS | -0.10 (0.01) | -0.002 (0.02) | 0.12 (0.02) | 0.07 (0.02)  ♂ 0.37 (0.42)  ♀ **0.75 (0.62)** | 0.39 (0.36)  ♂ -0.41 (0.03)  ♀ **0.94 (0.02)** | 0.25 (0.04) | **0.59 (0.06)** | 0.08 (0.03) | 0.29 (0.02) |
|  | HD-LPS | -0.07 (0.01) | 0.21 (0.02) | 0.25 (0.02) | 0.22 (0.02)  ♂ -0.28 (0.02)  ♀ **0.80 (0.02)** | 0.41 (0.34) | 0.15 (0.04) | 0.26 (0.06) | 0.36 (0.03) | 0.33 (0.02) |
| Anti-inflammatory index | LD-LPS | -0.17 (0.01) | -0.07 (0.20) | 0.05 (0.02) | 0.06 (0.02) | 0.13 (0.43) | 0.27 (0.05) | 0.33 (0.07) | 0.001 (0.04) | 0.19 (0.02) |
|  | HD-LPS | -0.16 (0.01)  ♂ 0.23 (0.01)  ♀ **-0.94 (0.01)** | 0.23 (0.02)  ♂ -0.37 (0.02)  ♀ 0.85 (0.02) | 0.21 (0.02)  ♂ -0.26 (0.02)  ♀ **0.79 (0.03)** | 0.23 (0.02)  ♂ -0.10 (0.03)  ♀ 0.52 (0.03) | 0.18 (0.39)  ♂ -0.07 (0.47)  ♀ 0.59 (0.76) | 0.008 (0.04)  ♂ -0.04 (0.05)  ♀ 0.53 (0.09) | -0.15 (0.07)  ♂ -0.54 (0.07)  ♀ 0.55 (0.12) | 0.16 (0.03)  ♂ -0.08 (0.03)  ♀ 0.47 (0.06) | 0.10 (0.02) |
| Ratio pro/anti inflammatory index | LD-LPS | 0.01 (0.06) | 0.13 (0.08) | 0.23 (0.09) | -0.03 (0.09) | 0.12 (1.79) | 0.30 (0.19) | 0.09 (0.31) | 0.05 (0.15) | 0.17 (0.08) |
|  | HD-LPS | **0.53 (0.11)** | -0.29 (0.16) | -0.35 (0.19) | **-0.54 (0.16)** | -0.39 (3.45) | -0.37 (0.38) | **-0.55 (0.57)** | -0.49 (0.27) | **-0.55 (0.14)** |
| TNF-α | LD-LPS | 0.007 (0.02) | -0.22 (0.03) | -0.04 (0.03) | -0.04 (0.03) | 0.16 (0.58) | 0.19 (0.06) | 0.50 (0.09) | -0.05 (0.05) | 0.18 (0.02) |
|  | HD-LPS | 0.17 (0.02) | 0.01 (0.02) | 0.06 (0.03) | -0.03 (0.02) | 0.12 (0.46) | -0.007 (0.05) | -0.07 (0.08) | 0.21 (0.04) | 0.11 (0.02) |
| IL-1β | LD-LPS | 0.009 (0.02) | -0.17 (0.03) | -0.05 (0.03) | 0.01 (0.03) | 0.34 (0.57) | 0.28 (0.06) | 0.49 (0.09) | -0.06 (0.05) | 0.21 (0.03) |
|  | HD-LPS | 0.03 (0.01) | 0.17 (0.01)  ♂ 1.06 (0.02)  ♀ -0.43 (0.02) | 0.21 (0.02)  ♂ 1.13 (0.02)  ♀ -0.32 (0.02) | 0.20 (0.02)  ♂ 0.89 (0.02)  ♀ -0.32 (0.02) | 0.36 (0.32)  ♂ 0.80 (0.61)  ♀ 0.20 (0.41) | 0.10 (0.04) | 0.16 (0.06) | 0.36 (0.03) | 0.28 (0.01) |
| IL-1RA | LD-LPS | -0.02 (0.009) | -0.16 (0.01)  **♂ 1.14 (0.009)**  ♀ -0.51 (0.01) | -0.009 (0.02)  **♂ 1.05 (0.009)**  ♀ -0.31 (0.02) | 0.009 (0.01)  ♂ 0.94 (0.009)  ♀ -0.37 (0.02) | 0.04 (0.28) | 0.31 (0.03) | 0.25 (0.05) | 0.11 (0.02) | 0.25 (0.01) |
|  | HD-LPS | 0.05 (0.007) | -0.07 (0.01)  ♂ 1.04 (0.005)  ♀ -0.64 (0.02) | 0.001 (0.01)  ♂ 0.92 (0.006)  ♀ -0.03 (0.02) | 0.09 (0.01) | 0.06 (0.22) | 0.16 (0.02) | -0.09 (0.04) | 0.11 (0.02) | 0.11 (0.009) |
| IL-4 | LD-LPS | - | - | - | - | - | - | - | - | - |
|  | HD-LPS | - | - | - | - | - | - | - | - | - |
| IL-10 | LD-LPS | -0.27 (0.01) | 0.05 (0.02) | 0.10 (0.02) | 0.10 (0.02) | 0.19 (0.43) | 0.15 (0.05) | 0.32 (0.07) | -0.11 (0.04) | 0.09 (0.02) |
|  | HD-LPS | -0.30 (0.01) | 0.43 (0.02) | 0.32 (0.02)  ♂ 0.60 (0.04)  ♀ | 0.28 (0.02) | 0.23 (0.39) | -0.04 (0.04) | -0.13 (0.07) | 0.15 (0.03) | 0.04 (0.02) |
| CCL2 | LD-LPS | -0.20 (0.01) | **0.49 (0.01)** | 0.24 (0.02) | 0.30 (0.02) | **0.45 (0.29)** | -0.14 (0.04) | 0.23 (0.06) | 0.18 (0.03) | 0.10 (0.01) |
|  | HD-LPS | -0.24 (0.01) | 0.15 (0.02) | 0.04 (0.02) | 0.21 (0.02) | 0.41 (0.39) | 0.13 (0.05) | **0.56 (0.06)** | -0.12 (0.03) | 0.13 (0.02)  ♂ 0.30 (0.03)  ♀0.04 (0.02) |
| CCL3 | LD-LPS | 0.02 (0.01) | -0.15 (0.02) | 0.04 (0.02) | -0.09 (0.02) | 0.33 (0.38) | 0.32 (0.04) | **0.55 (0.06)** | 0.06 (0.03) | 0.30 (0.02) |
|  | HD-LPS | 0.08 (0.009) | 0.14 (0.01) | 0.14 (0.02)  ♂ 0.76 (0.02)  ♀ -0.17 (0.02) | 0.08 (0.01)  ♂ 0.51 (0.02)  ♀ -0.21 (0.02) | 0.30 (0.27) | 0.05 (0.03) | 0.01 (0.05) | 0.38 (0.02) | 0.24 (0.01) |
| CCL4 | LD-LPS | -0.22 (0.01) | 0.04 (0.01)  **♂ 1.16 (0.01)**  ♀ -0.43 (0.02) | 0.25 (0.02) | 0.08 (0.02)  ♂ 0.88 (0.02)  ♀ -0.31 (0.02) | 0.18 (0.31) | 0.28 (0.03) | 0.43 (0.05) | 0.15 (0.03) | 0.31 (0.01) |
|  | 10ug/ml | -0.31 (0.007) | 0.29 (0.01)  ♂ 1.16 (0.006)  ♀ -0.19 (0.01) | 0.42 (0.01)  ♂ 1.03 (0.008)  ♀ 0.14 (0.02) | 0.32 (0.01)  ♂ 0.78 (0.01)  ♀ 0.05 (0.02) | 0.27 (0.22)  ♂ 0.73 (0.35)  ♀ 0.28 (0.28) | 0.26 (0.03)  ♂ 0.70 (0.04)  ♀ 0.30 (0.03) | 0.28 (0.04)  ♂ 0.73 (0.05)  ♀ 0.11 (0.05) | 0.46 (0.02)  ♂ 0.71 (0.03)  ♀ 0.23 (0.03) | 0.44 (0.009) |
| **Ex-vivo** | | | | | | | | | | |
| Inflammatory index |  | **0.54 (0.009)** | -0.30 (0.01) | -0.28 (0.02) | -0.29 (0.01) | -0.36 (0.29) | -0.13 (0.03) | -0.32 (0.04) | -0.24 (0.02) | -0.24 (0.01) |
| Pro-inflammatory index |  | **0.47 (0.008)** | -0.27 (0.01) | -0.21 (0.01) | -0.19 (0.01) | -0.29 (0.24) | -0.08 (0.02) | -0.18 (0.03) | -0.04 (0.02) | -0.10 (0.008) |
| Anti-inflammatory index |  | **0.49 (0.01)** | -0.27 (0.02) | -0.28 (0.02) | -0.29 (0.02) | -0.34 (0.41) | -0.14 (0.04) | -0.35 (0.06) | -0.31 (0.03) | -0.28 (0.01) |
| Ratio pro/anti inflammatory index |  | 0.31 (0.18) | -0.33 (0.22) | **-**0.35 (0.26) | **-0.46 (0.24)** | -0.09 (5.52) | 0.009 (0.52) | -0.11 (0.75) | -0.13 (0.37) | -0.10 (0.18) |
| hsCRP |  | 0.36 (0.01) | -0.27 (0.02) | -0.21 (0.02) | -0.18 (0.02) | -0.21 (0.43) | -0.07 (0.04) | -0.19 (0.06) | 0.01 (0.03) | -0.07 (0.02) |
| TNF-α |  | **0.47 (0.01)** | -0.24 (0.01) | -0.24 (0.01) | -0.15 (0.01) | -0.18 (0.24) | -0.17 (0.02) | -0.25 (0.03) | -0.29 (0.02*)* | -0.26 (0.01) |
| sTNF-R2 |  | **0.47 (0.004)** | -0.24 (0.01) | 0.23 (0.01) | -0.36 (0.01) | **-0.42 (0.11)** | -0.2 (0.01) | -0.38 (0.02) | -0.27 (0.07) | -0.30(0.004) |
| Il-1β |  | **-** | **-** | - | - | - | **-** | **-** | *-* | **-** |
| IL-1RA |  | 0.40 (0.01) | -0.24 (0.01) | -0.27 (0.01) | -0.17 (0.01) | -0.177 (0.21) | -0.04 (0.02) | -0.24 (0.28) | -0.28 (0.01) | 0.21 (0.01) |

Data represent standardized regression coefficient-β (standard error, unstandardized B) of various psychological factors demonstrating an association with several neuroimmune responses *ex-vivo* and after *in-vitro* whole blood stimulation using linear regression models. All neuroimmune responses are Ln-transformed. The *in-vitro* neuroimmune responses are normalized for monocyte count. Significant values are in bold font (^*^p < 0.05). TNF-α, tumor necrosis factor -α; IL-1β, interleukin -1β; IL-1RA, interleukin -receptor antagonist; IL-4, interleukin – 4; IL-10, interleukin -10; CCL2 / MCP1, c-c-motif ligand 2 also referred to as monocyte chemoattractant protein 1; CCL3 / MIP1α, c-c-motif ligand 3 also referred to as macrophage inflammatory protein 1α; CCL4 / MIP1β, c-c-motif ligand 4 also referred to as macrophage inflammatory protein 1β; TNFα : IL-10, ratio between TNFα : IL-10; IL-1β : IL-1RA, ratio between IL-1β : IL-1RA; 1ng/ml (LD-LPS), 1 milliliter whole-blood stimulation with 1 nanogram TLR4 agonist lipopolysacharide; 10µg/ml (HD-LPS), 1 milliliter whole blood stimulation with 10 microgram of TLR4 agonist lipopolysacharide; ♂ effect modification for males; ♀ effect modification for females.

^a^Refers to mental health inventory -5 score

^b^Refers to score on the depression, anxiety and stress scale 21 subsection stress

^c^Refers to the score on the depression, anxiety and stress scale 21 subsection depression

^d^Refers to the score on the depression, anxiety and stress scale 21 subsection anxiety

^e^Refers to total score on the tampa scale questionnaire higher than 37

^f^Refers to the total score on the pain catastrophising scale

**SUPPLEMENTARY H1** Association of lifestyle factors with neuroimmune responses in healthy participants

|  | | **Physical Activity^a^** | **Smoking^b^** | **Alcohol use^c^** | **BMI** | **Visceral Adipose^d^** |
| --- | --- | --- | --- | --- | --- | --- |
|  | | **Standardized- adjusted** β **(SE)** | **Standardized- adjusted** β **(SE)** | **Standardized- adjusted** β **(SE)** | **Standardized- adjusted** β **(SE)** | **Standardized- adjusted** β **(SE)** |
| **In-vitro** | | | | | | |
| Inflammatory index | LD-LPS | 0.04 (0.02) | 0.31 (0.58) | -0.17 (0.32) | **-0.40 (0.03)** | -0.20 (0.007) |
|  | HD-LPS | 0.14 (0.02) | **0.59 (0.54)** | -0.18 (0.32) | 0.08 (0.03) | -0.16 (0.007) |
| Pro-inflammatory index | LD-LPS | 0.04 (0.02) | 0.27 (0.60) | -0.17 (0.33) | **-0.40 (0.03)** | -0.22 (0.007) |
|  | HD-LPS | 0.10 (0.02) | 0.43 (0.57) | -0.24 (0.32) | 0.14 (0.03) | -0.12 (0.007) |
| Anti-inflammatory index | LD-LPS | 0.03 (0.02) | 0.37 (0.60) | -0.15 (0.35) | **-0.38 (0.03)** | -0.16 (0.007) |
|  | HD-LPS | 0.16 (0.03) | **0.74 (0.67)** | -0.003 (0.45) | -0.07 (0.04) | -0.18 (0.009) |
| Ratio pro/anti inflammatory index | LD-LPS | 0.34 (0.03) | -0.06 (1.07) | -0.18 (0.57) | 0.15 (0.06) | 0.19 (0.01) |
|  | HD-LPS | -0.26 (0.13) | -0.07 (4.46) | -0.10 (2.39) | -0.24 (0.23) | -0.40 (0.05) |
| TNF-α | LD-LPS | 0.07 (0.02) | 0.03 (0.73) | -0.10 (0.39) | **-0.42 (0.03)** | -0.19 (0.008) |
|  | HD-LPS | 0.11 (0.02) | 0.18 (0.64) | -0.27 (0.33) | **0.52 (0.03)** | -0.05 (0.007) |
| IL-1β | LD-LPS | -0.02 (0.04) | 0.14 (1.19) | -0.19 (0.63) | **-0.56 (0.05)** | -0.28 (0.01) |
|  | HD-LPS | 0.10 (0.02) | 0.32 (0.51) | -0.32 (0.26) | 0.21 (0.03) | 0.04 (0.006) |
| IL-1RA | LD-LPS | -0.003 (0.01) | 0.33 (0.42) | -0.12 (0.24)  ♂ 0.13 (0.22)  ♀ -0.71 (0.47) | **-0.40 (0.02)** | -0.12 (0.005) |
|  | HD-LPS | -0.02 (0.01) | 0.55 (0.29) | -0.05 (0.17) | -0.25 (0.02) | -0.05 (0.004) |
| IL-4 | LD-LPS | - | - | - | - | - |
|  | HD-LPS | **-** | **-** | **-** | **-** | **-** |
| IL-10 | LD-LPS | 0.06 (0.02) | **0.37 (0.59)** | -0.17 (0.34) | **-0.31 (0.03)** | -0.19 (0.007) |
|  | HD-LPS | 0.29 (0.02) | **0.64 (0.52)** | 0.04 (0.32) | 0.13 (0.03) | -0.23 (0.007) |
| CCL2 | LD-LPS | 0.03 (0.02) | **0.63 (0.63)** | -0.15 (0.37) | 0.31 (0.04) | 0.07 (0.008) |
|  | HD-LPS | -0.06 (0.02) | 0.30 (0.79) | 0.17 (0.43) | -0.39 (0.04) | 0.17 (0.009) |
| CCL3 | LD-LPS | 0.05 (0.02) | 0.13 (0.69) | -0.09 (0.38) | **-0.51 (0.03)** | -0.18 (0.008) |
|  | HD-LPS | 0.21 (0.02) | 0.27 (0.39) | -0.16 (0.21) | 0.11 (0.02) | -0.18 (0.004) |
| CCL4 | LD-LPS | 0.008 (0.01) | 0.11 (0.46) | -0.13 (0.24) | **-0.36 (0.02)** | -0.28 (0.005) |
|  | HD-LPS | -0.04 (0.009) | 0.19 (0.29) | -0.18 (0.15) | 0.005 (0.02) | **-0.37 (0.003)** |
| **Ex-vivo** | | | | | | |
| Inflammatory index |  | 0.24 (0.02) | 0.19 (0.54) | 0.08 (0.39) | 0.23 (0.04) | **0.52 (0.006)** |
| Pro-inflammatory index |  | 0.16 (0.02) | 0.10 (0.63) | -0.05 (0.44) | 0.37 (0.04) | 0.19 (0.41) |
| Anti-inflammatory index |  | 0.27 (0.02) | 0.25 (0.58) | 0.19 (0.41) | 0.04 (0.04) | 0.39 (0.007) |
| Ratio pro/anti inflammatory index |  | 0.34 (0.66) | **-0.61 (17.8)** | -0.14 (16.4) | -0.34 (1.40) | -0.14 (16.4) |
| hsCRP |  | -0.28 (0.02) | 0.11 (0.64) | 0.18 (0.47) | **0.47 (0.04)** | 0.40 (0.008) |
| TNF-α |  | 0.44 (0.01) | -0.08 (0.37) | -0.25 (0.25) | 0.10 (0.02) | 0.55 (0.004) |
| sTNF-R2 |  | **0.46 (0.005)** | -0.29 (0.17) | 0.21 (0.12) | -0.01 (0.01) | **0.49 (0.002)** |
| Il-1β |  | 0.33 (0.06) | 0.29 (2.52) | 0.64 (1.84) | 0.22 (0.27) | 0.31 (0.03) |
| IL-1RA |  | -0.05 (0.009) | **0.67 (0.22)** | 0.08 (0.21) | 0.07 (0.02) | 0.10 (0.004) |

Data represent standardized regression coefficient β (standard error, unstandardized B) of various lifestyle factors demonstrating an association with *ex-vivo* and *in-vitro* inflammatory indexes and of *in-vitro* and *ex-vivo* single inflammatory mediators using linear regression analysis. All single neuroimmune responses are ln-transformed. The *in-vitro* neuroimmune responses are normalized for monocyte count. Significant values are in bold font (^*^p < 0.05). TNF-α, tumor necrosis factor -α; IL-1β, interleukin -1β; IL-1RA, interleukin -receptor antagonist; IL-4, interleukin – 4; IL-10, interleukin -10; CCL2 / MCP1, c-c-motif ligand 2 also referred to as monocyte chemoattractant protein 1; CCL3 / MIP1α, c-c-motif ligand 3 also referred to as macrophage inflammatory protein 1α; CCL4 / MIP1β, c-c-motif ligand 4 also referred to as macrophage inflammatory protein 1β; TNFα : IL-10, ratio between TNFα : IL-10; IL-1β : IL-1RA, ratio between IL-1β : IL-1RA; 1ng/ml (LD-LPS), 1 milliliter whole-blood stimulation with 1 nanogram TLR4 agonist lipopolysacharide; 10µg/ml (HD-LPS), 1 milliliter whole blood stimulation with 10 microgram of TLR4 agonist lipopolysacharide; ♂ effect modification for males; ♀ effect modification for females.

^a^Refers to 1000/metabolic equivalent of a task

^b^Refers to current smoker, yes:1, no:0

^c^Refers to consuming alcohol, yes:1, no:0

^d^Refers to linear distance in millimeters between the posterior aspect of peritoneum and anterior aspect of lumbar vertebra T10

**SUPPLEMENTARY H2** Association of clinical factors with neuroimmune responses in healthy participants

|  | | **Pain intensity^a^** | **Disability^b^** | **Sex^c^** | **Central Sensitization^d^** | **Number of co-morbidities** | **Insomnia^d^** |
| --- | --- | --- | --- | --- | --- | --- | --- |
|  | | **Standardized- adjusted** β **(SE)** | **Standardized- adjusted** β **(SE)** | **Standardized- adjusted** β **(SE)** | **Standardized- adjusted** β **(SE)** | **Standardized- adjusted** β **(SE)** | **Standardized- adjusted** β **(SE)** |
| **In-vitro** | | | | | | | |
| Inflammatory index | LD-LPS | N/A | 0.14 (0.02) | 0.21 (0.28) | 0.18 (0.45) | **-0.42 (0.29)** | 0.04 (0.08) |
|  | HD-LPS | N/A | 0.32 (0.02) | 0.23 (0.28) | 0.33 (0.43) | **-0.52 (0.30)** | 0.12 (0.08) |
| Pro-inflammatory index | LD-LPS | N/A | 0.14 (0.02) | 0.17 (0.28) | 0.16 (0.45) | **-0.45 (0.29)** | 0.02 (0.08) |
|  | HD-LPS | N/A | 0.27 (0.02) | 0.08 (0.29) | 0.24 (0.44) | **-0.49 (0.31)** | 0.06 (0.08) |
| Anti-inflammatory index | LD-LPS | N/A | 0.12 (0.02) | 0.27 (0.29) | 0.21 (0.47) | **-0.35 (0.34)** | 0.08 (0.08) |
|  | HD-LPS | N/A | 0.32 (0.02) | **0.47 (0.34)** | 0.41 (0.56) | **-0.43 (0.45)** | 0.19 (0.10) |
| Ratio pro/anti inflammatory index | LD-LPS | N/A | 0.06 (0.03) | -0.14 (0.50) | 0.06 (0.80) | 0.21 (0.64) | 0.05 (0.14) |
|  | HD-LPS | N/A | -0.02 (0.12) | 0.17 (2.08) | -0.08 (3.33) | **-0.68 (1.97)** | -0.16 (0.56) |
| TNF-α | LD-LPS | N/A | 0.003 (0.02) | 0.18 (0.33) | 0.03 (0.55) | -0.23 (0.42) | -0.08 (0.09) |
|  | HD-LPS | N/A | 0.25 (0.02) | -0.06 (0.31) | 0.26 (0.47) | -0.20 (0.39) | -0.13 (0.08) |
| IL-1β | LD-LPS | N/A | 0.15 (0.03) | 0.32 (0.53) | 0.17 (0.88) | **-0.39 (0.64)** | 0.09 (0.15) |
|  | HD-LPS | N/A | 0.32 (0.01) | 0.03 (0.25) | 0.27 (0.37) | -0.27 (0.31) | 0.19 (0.07) |
| IL-1RA | LD-LPS | N/A | 0.06 (0.01) | 0.35 (0.19) | 0.15 (0.33) | -0.25 (0.26) | 0.07 (0.06) |
|  | HD-LPS | N/A | 0.21 (0.008) | **0.50 (0.13)** | 0.29 (0.23) | -0.28 (0.19) | 0.23 (0.04) |
| IL-4 | LD-LPS | - | **-** | - | - | - | - |
|  | HD-LPS | - | **-** | - | - | **-** | - |
| IL-10 | LD-LPS | N/A | 0.17 (0.02) | 0.16 (0.30) | 0.24 (0.45) | **-0.41 (0.28)** | 0.08 (0.08) |
|  | HD-LPS | N/A | 0.31 (0.02) | 0.25 (0.27) | 0.36 (0.42) | -0.41 (0.33) | 0.08 (0.08) |
| CCL2 | LD-LPS | N/A | 0.27 (0.02) | -0.27 (0.32) | 0.25 (0.51) | **-0.47 (0.38)** | 0.13 (0.09) |
|  | HD-LPS | N/A | 0.06 (0.02) | -0.08 (0.39) | 0.002 (0.61) | -0.33 (0.47) | 0.04 (0.10) |
| CCL3 | LD-LPS | N/A | 0.09 (0.02) | 0.23 (0.31) | 0.10 (0.52) | -0.29 (0.39) | 0.02 (0.09) |
|  | HD-LPS | N/A | 0.23 (0.01) | 0.15 (0.19) | 0.16 (0.29) | -0.32 (0.23) | 0.15 (0.05) |
| CCL4 | LD-LPS | N/A | 0.03 (0.01) | 0.20 (0.21) | 0.06 (0.35) | **-0.35 (0.23)** | -0.06 (0.06) |
|  | HD-LPS | N/A | -0.16 (0.03) | 0.001 (0.008) | 0.21 (0.13) | **-0.42 (0.14)** | 0.06 (0.22) |
| **Ex-vivo** | | | | | | | |
| Inflammatory index |  | N/A | 0.21 (0.02) | -0.13 (0.30) | 0.21 (0.52) | 0.19 (0.43) | -0.40 (0.08) |
| Pro-inflammatory index |  | N/A | 0.24 (0.02) | -0.16 (0.34) | 0.19 (0.59) | 0.25 (0.49) | -0.39 (0.09) |
| Anti-inflammatory index |  | N/A | 0.14 (0.02) | -0.06 (0.33) | 0.19 (0.56) | 0.10 (0.47) | -0.34 (0.09) |
| Ratio pro/anti inflammatory index |  | N/A | 0.10 (0.81) | -0.20 (12.64) | 0.20 (0.59) | -0.12 (18.6) | 0.12 (3.69) |
| hsCRP |  | N/A | 0.16 (0.02) | -0.001 (0.37) | 0.03 (0.64) | 0.33 (0.51) | -0.27 (0.10) |
| TNF-α |  | N/A | 0.27 (0.01) | -0.22 (0.20) | 0.29 (0.34) | 0.10 (0.29) | -0.29 (0.06) |
| sTNF-R2 |  | N/A | -0.09 (0.006) | -0.31 (0.09) | -0.15 (0.17) | 0.14 (0.14) | -0.35 (0.03) |
| Il-1β |  | N/A | -0.34 (0.15) | -0.39 (1.45) | N/A | 0.64 (1.84) | -0.02 (1.10) |
| IL-1RA |  | N/A | 0.30 (0.01) | 0.21 (0.16) | **0.44 (0.25)** | 0.01 (0.23) | -0.16 (0.05) |

Data represent standardized regression coefficient β (standard error, unstandardized B) of various clinical factors demonstrating an association with *ex-vivo* and *in-vitro* inflammatory indexes and of *in-vitro* and *ex-vivo* single inflammatory mediators using linear regression analysis. All single neuroimmune responses are ln-transformed. The *in-vitro* neuroimmune responses are normalized for monocyte count. Significant values are in bold font (^*^p < 0.05) TNF-α, tumor necrosis factor -α; IL-1β, interleukin -1β; IL-1RA, interleukin -receptor antagonist; IL-4, interleukin – 4; IL-10, interleukin -10; CCL2 / MCP1, c-c-motif ligand 2 also referred to as monocyte chemoattractant protein 1; CCL3 / MIP1α, c-c-motif ligand 3 also referred to as macrophage inflammatory protein 1α; CCL4 / MIP1β, c-c-motif ligand 4 also referred to as macrophage inflammatory protein 1β; TNFα : IL-10, ratio between TNFα : IL-10; IL-1β : IL-1RA, ratio between IL-1β : IL-1RA; 1ng/ml (LD-LPS), 1 milliliter whole-blood stimulation with 1 nanogram TLR4 agonist lipopolysacharide; 10µg/ml (HD-LPS), 1 milliliter whole blood stimulation with 10 microgram of TLR4 agonist lipopolysacharide; ♂ effect modification for males; ♀ effect modification for females.

^a^Refers to pain intensity measured on the visual analogue scale, 0-100

^b^Refers to score on the neck disability index, 0-100

^c^Male:0 Female:1

^d^Refers to total score on the central sensitisation questionnaire higher than 40

^e^Refers having insomnia (pittsburg sleep quality index score above 5)

**SUPPLEMENTARY H3** Association of psychological factors with neuroimmune responses in healthy partciipants

|  | | **Mental Health^a^** | **Stress^b^** | **Depression^c^** | **Anxiety^d^** | **Kinesiophobia^e^** | **Pain rumination** | **Pain magnification** | **Pain helplessness** | **Catastrophizing^f^** |
| --- | --- | --- | --- | --- | --- | --- | --- | --- | --- | --- |
|  | | **Standardized- adjusted** β **(SE)** | **Standardized- adjusted** β **(SE)** | **Standardized- adjusted** β **(SE)** | **Standardized- adjusted** β **(SE)** | **Standardized- adjusted** β **(SE)** | **Standardized- adjusted** β **(SE)** | **Standardized- adjusted** β **(SE)** | **Standardized- adjusted** β **(SE)** | **Standardized- adjusted** β **(SE)** |
| **In-vitro** | | | | | | | | | | |
| Inflammatory index | LD-LPS | **-0.33 (0.01)** | 0.26 (0.02) | -0.05 (0.09) | 0.24 (0.06) | 0.27 (0.58) | -0.13 (0.03) | -0.10 (0.05) | -0.03 (0.04) | -0.10 (0.01) |
|  | HD-LPS | **-0.42 (0.01)** | 0.20 (0.02) | 0.04 (0.09) | -0.02 (0.06) | 0.19 (0.62) | -0.18 (0.03) | -0.19 (0.05) | -0.17 (0.04) | -0.19 (0.01) |
| Pro-inflammatory index | LD-LPS | **-0.35 (0.01)** | 0.24 (0.02) | -0.06 (0.10) | 0.23 (0.06) | 0.31 (0.58) | -0.15 (0.03) | -0.13 (0.05) | -0.03 (0.04) | -0.12 (0.01) |
|  | HD-LPS | **-0.32 (0.01)** | 0.08 (0.02) | 0.003 (0.09) | -0.04 (0.06) | 0.18 (0.62) | -0.14 (0.03) | -0.19 (0.05) | -0.15 (0.04) | -0.17 (0.01) |
| Anti-inflammatory index | LD-LPS | -0.28 (0.01) | 0.28 (0.02) | -0.02 (0.10) | 0.25 (0.06) | 0.17 (0.66) | -0.07 (0.03) | -0.03 (0.06) | -0.02 (0.05) | -0.05 (0.02) |
|  | HD-LPS | **-0.49 (0.02)** | 0.38 (0.03) | 0.11 (0.13) | 0.03 (0.08) | 0.16 (0.84) | -0.21 (0.03) | -0.14 (0.07) | -0.16 (0.06) | -0.19 (0.02) |
| Ratio pro/anti inflammatory index | LD-LPS | 0.06 (0.02) | **-0.49 (0.03)** | -0.03 (0.17) | -0.20 (0.10) | **-0.48 (0.97)** | -0.15 (0.05) | -0.05 (0.09) | -0.33 (0.07) | 0.06 (0.03) |
|  | HD-LPS | -0.12 (0.10) | 0.09 (0.14) | -0.46 (0.61) | 0.05 (0.44) | **0.88 (2.22)** | -0.25 (0.18) | -0.05 (0.37) | -0.02 (0.31) | -0.15 (0.10) |
| TNF-α | LD-LPS | -0.27 (0.02) | 0.04 (0.02) | -0.10 (0.11) | 0.001 (0.07) | 0.10 (0.75) | -0.25 (0.03) | -0.15 (0.06) | -0.008 (0.05) | -0.17 (0.02) |
|  | HD-LPS | -0.10 (0.02) | -0.08 (0.02) | 0.14 (0.10) | -0.20 (0.06) | -0.11 (0.67) | 0.12 (0.03) | -0.06 (0.05) | -0.002 (0.05) | 0.05 (0.02) |
| IL-1β | LD-LPS | -0.24 (0.03) | 0.32 (0.04) | -0.02 (0.19) | 0.34 (1.13) | -0.21 (0.05) | -0.19 (0.10) | -0.08 (0.08) | -0.19 (0.03) | -0.003 (0.01) |
|  | HD-LPS | -0.08 (0.01) | -0.07 (0.02) | 0.05 (0.08) | -0.06 (0.05) | -0.09 (0.54) | -0.11 (0.02) | -0.17 (0.04) | 0.16 (0.04) | -0.15 (0.01) |
| IL-1RA | LD-LPS | 0.23 (0.01) | 0.33 (0.01) | -0.03 (0.07) | 0.27 (0.04) | 0.08 (0.45) | -0.10 (0.02) | -0.04 (0.04) | 0.04 (0.03) | -0.05 (0.01) |
|  | HD-LPS | -0.13 (0.007)  ♂ -0.05 (0.006)  ♀ -0.17 (0.02) | 0.32 (0.01) | -0.12 (0.05) | 0.22 (0.03) | 0.12 (0.33) | -0.24 (0.01) | -0.08 (0.03) | -0.11 (0.02) | -0.18 (0.007) |
| IL-4 | LD-LPS | - | - | - | - | - | - | - | - | - |
|  | HD-LPS | - | - | - | - | - | - | - | - | - |
| IL-10 | LD-LPS | **-0.30 (0.01)** | 0.19 (0.02) | -0.01 (0.10) | 0.20 (0.06) | 0.23 (0.63) | -0.04 (0.03) | -0.02 (0.06) | -0.08 (0.05) | -0.05 (0.02) |
|  | HD-LPS | **-0.66 (0.01)** | 0.29 (0.02) | 0.29 (0.09) | -0.17 (0.06) | 0.13 (0.61) | -0.10 (0.03) | -0.14 (0.05) | -0.15 (0.04) | -0.14 (0.01) |
| CCL2 | LD-LPS | -0.31 (0.02) | 0.19 (0.02) | -0.02 (0.11) | 0.18 (0.07) | 0.26 (0.70) | 0.22 (0.03) | 0.14 (0.06) | 0.04 (0.05) | 0.17 (0.02) |
|  | HD-LPS | -0.36 (0.02) | 0.28 (0.03) | 0.34 (0.08) | 0.39 (0.76) | -0.19 (0.03) | -0.04 (0.07) | 0.08 (0.06) | -0.09 (0.02) | -0,09 (0.01) |
| CCL3 | LD-LPS | -0.29 (0.01) | 0.22 (0.02) | -0.02 (0.11) | 0.24 (0.07) | 0.19 (0.69) | -0.24 (0.03) | -0.19 (0.06) | -0.07 (0.05) | -0.19 (0.01) |
|  | HD-LPS | -0.33 (0.008) | 0.04 (0.01) | 0.13 (0.06) | -0.10 (0.04) | 0.05 (0.41) | -0.25 (0.02) | -0.29 (0.03) | -0.30 (0.03) | -0.30 (0.008) |
| CCL4 | LD-LPS | -0.23 (0.01) | 0.17 (0.01) | -0.05 (0.07) | 0.13 (0.05) | **0.30 (0.41)** | -0.11 (0.02) | -0.09 (0.04) | -0.01 (0.03) | -0.09 (0.01) |
|  | HD-LPS | -0.06 (0.04) | -0.15 (0.006) | -0.02 (0.05) | -0.11 (0.03) | **0.33 (0.26)** | -0.006 (0.01) | -0.04 (0.02) | -0.07 (0.02) | -0.04 (0.006) |
| **Ex-vivo** | | | | | | | | | | |
| Inflammatory index |  | **0.44 (0.01)** | -0.11 (0.02) | -0.21 (0.10) | 0.11 (0.07) | -0.03 (0.74) | 0.04 (0.03) | -0.03 (0.06) | 0.25 (0.05) | 0.10 (0.02) |
| Pro- inflammatory index |  | **0.53 (0.02)** | -0.28 (0.03) | -0.23 (0.12) | -0.01 (0.08) | 0.01 (0.83) | -0.04 (0.04) | -0.07 (0.07) | 0.22 (0.05) | 0.03 (0.02) |
| Anti-inflammatory index |  | 0.26 (0.02) | 0.08 (0.02) | -0.15 (0.11) | 0.21 (0.07) | -0.07 (0.79) | 0.12 (0.03) | 0.02 (0.07) | 0.24 (0.05) | 0.14 (0.02) |
| Ratio pro/anti inflammatory index |  | -0.08 (0.64) | 0.12 (0.96) | 0.05 (4.54) | 0.19 (2.78) | -0.04 (31.3) | 0.07 (1.33) | 0.18 (2.53) | 0.08 (2.05) | 0.11 (0.68) |
| hsCRP |  | 0.33 (0.02) | -0.28 (0.03) | -0.23 (0.12) | -0.17 (0.08) | 0.04 (0.89) | -0.12 (0.04) | -0.20 (0.07) | 0.19 (0.06) | -0.05 (0.02) |
| TNF-α |  | 0.39 (0.009) | -0.12 (0.02) | -0.04 (0.07) | 0.12 (0.05) | 0.13 (0.36) | 0.15 (0.02) | 0.09 (0.04) | 0.23 (0.03) | 0.18 (0.01) |
| sTNF-R2 |  | 0.35 (0.005) | -0.11 (0.007) | -0.27 (0.03) | 0.02 (0.02) | 0.04 (0.12) | -0.12 (0.01) | 0.02 (0.02)  ♂ **-0.50 (0.02)**  ♀ 0.23 (0.04) | 0.12 (0.02) | -0.1 (0.005) |
| Il-1β |  | 0.65 (0.08) | 0.21 (0.10) | -0.05 (0.87) | 0.15 (0.30) | 0.03 (0.26) | 0.03 (0.28) | 0.16 (0.41) | -0.31 (0.37) | -0.05 (0.15) |
| IL-1RA |  | 0.04 (0.008) | 0.23 (0.01) | 0.04 (0.06) | 0.29 (0.03) | 0.28 (0.02) | 0.30 (0.02) | 0.005 (0.03) | 0.23 (0.03) | 0.23 (0.03) |

Data represent standardized regression coefficient-β (standard error, unstandardized B) of various psychological factors demonstrating an association with several neuroimmune responses *ex-vivo* and after *in-vitro* whole blood stimulation using linear regression models. All neuroimmune responses are Ln-transformed. The *in-vitro* neuroimmune responses are normalized for monocyte count. Significant values are in bold font (^*^p < 0.05). TNF-α, tumor necrosis factor -α; IL-1β, interleukin -1β; IL-1RA, interleukin -receptor antagonist; IL-4, interleukin – 4; IL-10, interleukin -10; CCL2 / MCP1, c-c-motif ligand 2 also referred to as monocyte chemoattractant protein 1; CCL3 / MIP1α, c-c-motif ligand 3 also referred to as macrophage inflammatory protein 1α; CCL4 / MIP1β, c-c-motif ligand 4 also referred to as macrophage inflammatory protein 1β; TNFα : IL-10, ratio between TNFα : IL-10; IL-1β : IL-1RA, ratio between IL-1β : IL-1RA; 1ng/ml (LD-LPS), 1 milliliter whole-blood stimulation with 1 nanogram TLR4 agonist lipopolysacharide; 10µg/ml (HD-LPS), 1 milliliter whole blood stimulation with 10 microgram of TLR4 agonist lipopolysacharide; ♂ effect modification for males; ♀ effect modification for females.

^a^Refers to mental health inventory -5 score

^b^Refers to score on the depression, anxiety and stress scale 21 subsection stress

^c^Refers to the score on the depression, anxiety and stress scale 21 subsection depression

^d^Refers to the score on the depression, anxiety and stress scale 21 subsection anxiety

^e^Refers to total score on the tampa scale questionnaire higher than 37

^f^Refers to the total score on the pain catastrophizing scale
